# Can Substitutes Reduce Future Demand for Wildlife Products: A Case Study of China’s Millennial Generation

**DOI:** 10.1007/s10745-021-00279-0

**Published:** 2021-11-06

**Authors:** Katherine I. Rock, Douglas C. MacMillan

**Affiliations:** Durrell Institute of Conservation and Ecology (DICE), University of Kent, Canterbury, Kent, CT2 7NR UK

**Keywords:** China, Millennial, Wildlife product, Substitute, Behaviour change

## Abstract

China is one of the world’s leading consumer markets for wildlife products, yet there is little understanding of how demand will change in the future. In this study, we investigate the consumptive habits and attitudes of the millennial ‘*Juilinghou*’ demographic – a subset of society in China with the potential to substantially influence future demand for wildlife products. We surveyed 350 Chinese university students across Harbin and Beijing, China, and found that the intended future consumption of wildlife products was relatively low in this population but with a strong orientation towards wildlife products with medicinal properties. Seventy percent of those respondents who had used and/or intended to use wildlife products were willing to try substitutes, but this was heavily dependent on their price (cheaper) and quality. The insights gained through this survey are intended to meaningfully inform future initiatives to introduce sustainable substitutability into wildlife markets to alert future wildlife product consumers to alternative choices.

**Supplementary Information:**

The online version contains supplementary material available at 10.1007/s10745-021-00279-0.

## Introduction

China’s growing economic prosperity post-Maoist era (Whyte, 2012), compounded by the nation’s sustained population expansion making it the long-standing most populous country in the world (The World Bank, 2019), has rendered wildlife products accessible to an unprecedented potential market of consumers (Challender *et al.,* 2015). China’s ‘millennial’ generation, including the Juilinghou (九零后), born between 1990 to 2000 (Sheng, 2016; Yang, 2016) are a crucial group representing China’s future consumptive trends (Wang & Xu, 2009). Numbering around 415 million, equivalent to the total population of the United States (Fund Business Intelligence China, 2017), Chinese millennials have much higher purchasing power than previous generations (Zhang *et al.,* 2008) and as emergent independent consumers, could exert unprecedented pressure on rapidly declining wild animal populations.

Although prohibitive actions such as trade bans and enforcement measures are the dominant approach to reducing consumption in the Asian market (Challender & MacMillan, 2014), consumer-centric demand interventions promoting sustainable trade are gaining traction in the policy landscape (Broad & Burgess, 2016; Fabinyi & Liu, 2014). Product substitution may therefore have a distinct role to play in redirecting demand away from wild populations of conservation concern, to more sustainable alternatives (Moorhouse *et al.,* 2020), especially in China where efficacy and price of the product is a major consideration for most wildlife products (Broad & Burgess, 2016; Liu *et al.,* 2016). Indeed, Moorhouse *et al.* (2020) notes an increasing willingness among Chinese consumers to choose substitutes to protect endangered wildlife. For example, in the case of shark fin soup, consumers have shown a willingness to switch to sea cucumbers (Fabinyi, 2012; Fabinyi & Liu, 2014) including as a replacement dish in traditional banquets (Globe Scan, 2018). However, candidate wildlife product commodities of a similar perceived utility like sea cucumber, as well as other popular shark substitutes such as fish maw, swifts’ nest and Chinese caterpillar fungus, can themselves be tied with unsustainable harvest concerns (Ho & Shea, 2016). So, one would need to be conscious to not simply shift the burden of demand from sharks to another depleting species. One approach to this, as demonstrated by Farah and Boyce’s (2015) equilibrium bioeconomic model, is through supplying a non-renewable, extinct species substitute. Farah & Boyce’s model theoretically and empirically postulates that the presence of mammoth ivory would lower the demand for elephant ivory (more so than if a substitute was altogether absent), thus reducing extraction rates of elephants. The presence of a substitute may consequently lower the minimum viable population level at which extinction would ensue.

According to Broad and Burgess (2016), ‘alternatives’ that could alleviate poaching rates on wild supplies include the following: (1) products of the same species but sourced from an alternate supply country (i.e. where the species is more abundant); (2) products cultivated through artificial propagation or captive-breeding of non-domesticated species; (3) products of an alternative wildlife species with a similar utility; (4) products of domesticated animals or plant species with a similar utility; (5) products of inorganic or synthetic origin with a similar utility.

The purpose of this survey was to elicit a more culturally informed and nuanced understanding of the attitudes Chinese university students hold towards the usage of wildlife products and their associated substitutes, including a deeper understanding of the characteristics of wildlife products these potential buyers attribute most value to (Hinsley *et al.,* 2015). Our aims were therefore to:

- (1) Gauge the prevalence and type of wildlife consumption amongst Chinese university students.
- (2) Identify the motivations for and against consuming wildlife products.
- (3) Determine the extent to which students are familiar with and prepared to try substitutes.

Previous research suggests that wildlife consumption among young, better educated individuals with higher incomes is relatively high (Wasser & Jiao, 2010), with Zhang and Yin (2014) and Zhang *et al.* (2008) pinpointing males of this description as especially predisposed to consume wild animal products more frequently. Moreover, Meijer *et al.* (2017) found the younger demographic (defined as 18–30 years old) displayed the highest purchasing persistence towards elephant ivory products across fifteen Chinese cities compared to all other age groups. Hence, an important focus of this study was to investigate demand from the emergent younger generation, specifically Chinese millennials enrolled at university in the north east of China — a comparatively understudied region. Even more so, the significance of this study also extends to the viability of substitution to help fulfil demand for wildlife products, particularly in the wake of the COVID-19 global pandemic given the virus’s purported links to wildlife trade.

This study fundamentally explores the human dimensions to wildlife consumption, since human behaviour is the most significant driver of demand, to help understand how behavioural change can be accomplished in wildlife trade policy and practice (Wallen & Daut, 2017). In doing so, we introduce originality and innovative thinking to the existing portfolio of wildlife consumer market research. Specifically, we focus on the future role of the emergent Chinese millennial generation in the context of wildlife use, updating old perspectives about demand for wildlife products, while contributing to the discourse surrounding alternative ways to satiate fulfil demand for wildlife products, and to this end, the potential for future uptake of substitutes in this demographic.

## Material and Methods

### Study Sites

Our survey was drawn from Chinese University students. Data were collected through a structured questionnaire survey (see Appendix B), administered to a sampling frame consisting of students at four Chinese urban universities, two located in Harbin: (Northeast Forestry University, *n* = 205 and Heilongjiang University of Chinese Medicine, *n* = 40) and two in Beijing (Beijing University of Chemical Technology, *n* = 65 and Beijing University of Chinese Medicine, *n* = 40).

Harbin, the capital of China’s most North-eastern province Heilongjiang, and ranked as China’s tenth largest city (Chen *et al.,* 2013), was chosen as our primary study area due to: (1) its high concentration of young, educated people owing to this city’s numerous universities; (2) its geographical and historical links with Russian trade in the Far East (Wei, 2005) with the Heilongjiang province historically a centre for wildlife trade, due to hunting along the Amur-Ussuri river/Amur-Heilong transborder regions connecting far north China and Siberia, for example poaching of the Amur tiger and Amur leopard for their fur, as well as harvesting of wild ginseng (Simonov & Dahmar, 2008); (3) Harbin’s ‘second-tier’ city status, with a lower per capita GDP (Wasser & Jiao, 2010). Harbin was also highlighted as an important target for future communication efforts regarding wildlife consumption (Wasser & Jiao, 2010). Beijing, the capital city of the People’s Republic of China, was selected as our secondary study site for its metropolitan ‘first-tier city’ prominence, rendering it somewhat of a cultural 'trend-setter' in consumption preferences (Fabinyi & Liu, 2014).

### Sampling Methodology

Research ethics clearance was obtained from the University of Kent Research Ethics Advisory Group. Data gathering took place over a six-week period in June and July 2019, during which time a convenience sampling method was employed to circulate questionnaires in-person to respondents within our four chosen campus locations, typically in a classroom setting (outside of teaching hours) for ease of use, in order to generate the respondent set (Newing, 2010). A total of 358 questionnaires were issued, of which 350 were returned with valid responses; yielding a sex ratio somewhat biased towards males who constituted exactly 53% of survey respondents, compared to 47% female. Purposive sampling was also employed (Newing, 2010) to ensure all participants were studying at one of the four aforementioned universities, were of Chinese citizenship, and were born between 1989 – 2001 (within the age range 18 – 30 years old at the time of the survey), with a mean age of 21.8 years (see appendix A), all prerequisites for inclusion. Considering the caveats to this non — probabilistic approach, data collection was stratified across different times of day during weekdays and weekends in order to maximise the variety of respondents’ perspectives captured. We also used quota sampling to stratify our sample by sex, different years in university (randomly selected classes from each grade), and different disciplines, in order to minimize possible bias, checking the demographic characteristics of the respondent sample weekly (Doughty *et al.,* 2019).

The questionnaire was translated into Mandarin Chinese by a native speaker at Northeast Forestry University studying a discipline closely related to our research. The principal researcher was accompanied by a native speaking research assistant to allow for verbal exchange between translator and respondents, and to ensure the questionnaires were completed without errors (Newing, 2010). Due to this personal mode of administration, once each respondent had given their verbal and informed consent to partake in the survey having had the opportunity to ask any questions, they were given privacy so as not to feel pressured by the researchers’ presence. However, researchers were still within the vicinity to answer any further queries, since the survey was to be completed at the time of distribution. It was thought this approach would mitigate the probability of social desirability bias manifesting in the results; whereby the respondent over-reports socially ‘desirable’ behaviours or disguises what is are presumed less desirable attitudes in order to abide with social norms, based on their perceptions of what they thought we would want to hear (De Vaus, 2013).

### Questionnaire Design – Measures of Key Constructs

Following a small-scale pilot testing in-country, the survey was refined, according to feedback, to ensure questions were appropriately phrased and formatted for our target audience, rather than grounded in the perspective of a ‘western’ paradigm (Doughty *et al.,* 2019; Drury *et al.,* 2011a). The questionnaire consisted of 35 questions, a combination of open-ended and closed format questions, designed to collect both quantitative and qualitative data, the latter allowing the recording of subtleties that might otherwise have been overlooked (thus enhancing internal validity) (Drury *et al.,* 2011b). Likert scales were used as the main data collection instrument to measure latent constructs, namely attitudes, and perceived value, gathering data ordinal in nature (Likert, 1932).

Questions were split into four sections with the following objectives in mind:

- (1) understand respondents’ affinity for wildlife products through investigating their past consumption and stated relative order of preference for wildlife products serving different purposes;
- (2) assess the relative appeal of different attributes of wildlife products in order to glean insight into what motivations underpin the desire to consume — as well as determinants for not using — wildlife products;
- (3) establish the familiarity and readiness to try substitutes amongst Chinese students;
- (4) gather participant socio-demographic information to identify any correlates associated with consumption in this population.

### Statistical Analysis

Questionnaires were included in the analysis if participants had completed the demographic section. Questionnaire responses were coded through assigning numerical labels, open-ended responses collapsed into themes to also yield quantifiable data. The data were collated using Microsoft Excel, generating exploratory descriptive statistics to visualise and scrutinise the dataset. Inferential statistical analysis was performed using IBM SPSS v. 24 software. The level of measurement of all variables in the survey (except a ratio ‘age’ outcome) were categorical, generating nominal and ordinal data. As such, this justified the use of non-parametric tests that don’t assume underlying data normality. Crosstabulation and Pearson’s chi-squared test of independence (*X*^*2*^) were deployed for frequency testing and to analyse if demographic variables have any association with wildlife product and substitute — usage or non-usage — behaviour. Kruskall-Wallis *H* tests comparing multiple independent groups on an ordinal scale were also conducted (Kruskall & Wallis, 1952).

## Results

### Scale of Consumption

Over three-quarters of total respondents (78%, *n* = 269 of 350 participants) reported not yet using any wildlife products, with only a minority of this non – consumer group expressing an interest in doing so in the future (*n* = 38/269, 14% Future Users). Of those who had stated already having consumed a wildlife product (remaining 22%, *n* = 77/350), a greater proportion no longer wished to continue using (58% Former Users, *n* = 45/77). Notably, no respondents reported consuming wildlife products on a regular basis, including in the last six months.

Participants' likelihood of using wildlife products (hereafter their ‘affinity’), determined by both their reported past — and intended future — usage, based on socio-demographic characteristics were explored using a chi-square test (Table 1). Region of Origin and Programme of Study were the only significant variables, with respondents originating from north-east China having a significantly higher affinity (Table 1, + 2.2 z-adjusted residual score) i.e., a significantly larger proportion of individuals who had used wildlife products and intended to use them in the future (16.3%). Respondents in the ‘Life and Physical Sciences’ programme of study category had significantly more cases of Former Users, and Future Users, whereas respondents studying ‘Social Science and Humanities’ had a significantly higher proportion of individuals who were Non-Users (84%) compared to ‘Life and Physical Sciences’ (53%) (Table 1). Tests for respondent’s affinity to wildlife products dependant on their sex, place of study, year of study, urban or rural hometown, income bracket and whether or not they’d travelled outside of China were not significant.

**Table 1:** The relationship between respondent’s socio-demographic profile and their affinity for wildlife products

Explanatory variable **Respondent’s characteristics**		**Categories of affinity for wildlife products**						
		**Past & Future User**	**Former User**	**Future User**	**Non – User**	N	X^2^	P value
**Sex**	Male	23 (*13%*)	25 (*14%*)	21 (*12%*)	112 (*62%*)	181	6.4	0.168
	Female	9 (*6%*)	20 (*13%*)	15 (*9%*)	116 (*73%*)	160		
**Place of study**	Harbin	26 (*11%*)	34 (*14%*)	32 (*13%*)	151 (*62%*)	243	6.83	0.078
	Beijing	6 (*6%*)	11 (*10%*)	8 (*8%*)	80 (*76%*)	105		
**Region of origin in China**	North	7 (*8%*)	7 (*8%*)	14 (*16%*)	61 (*69%*)	89	23.09	0.027**
	North-east	**14 (*16%*)** ^**a. +2.2**^	11 (*13%*)	6 (*7%*)	55 (*64%*)	86		
	Eastern	2 (*4%*)	10 (*19%*)	10 (*19%*)	30 (*58%*)	52		
	Western	1 (*4%*)	1 (*4%*)	2 (*9%*)	19 (*83%*)	23		
	Central-south	7 (*13%*)	11 (*21%*)	3 (*6%*)	32 (*60%*)	53		
**Year of study**	1^st^ & 2^nd^ year	13 (*12%*)	16 (*15%*)	7 (*6%*)	72 (*67%*)	108	19.95	0.174
	3^rd^ & 4^th^ year	10 (*7%*)	16 (*11%*)	22 (*15%*)	96 (*67%*)	144		
	Postgraduate	9 (*10%*)	12 (*14%*)	10 (*11%*)	56 (*64%*)	87		
**Travelled outside of China**	YES	13 (*15%*)	11 (*13%*)	6 (*7%*)	54 (*64%*)	84	6.69	0.083
	NO	18 (*7%*)	31 (*12%*)	33 (*13%*)	167 (*67%*)	249		
**Type of hometown**	Urban	28 (*11%*)	35 (*14%*)	24 (*9%*)	170 (*66%*)	257	8.84	0.183
	Rural	3 (*5%*)	7 (*11%*)	12 (*19%*)	42 (*66%*)	64		
**Programme of Study**	Life & Physical Sciences	10 (*10%*)	**20 (*19%*)**^**a. +2.1**^	**19 (*18%*)**^**a. +2.9**^	**56(*53%*)**^**b. – 3.4**^	105	21.44	0.011**
	Engineering & Technology	15 (*13%*)	13 (*11%*)	8 (*7%*)	80 (*69%*)	116		
	Social Science & Humanities	2 (*4%*)	3 (*6%*)	3 (*6%*)	**43 (*84%***)^**a. + 3.0**^	51		
	Medicine	5 (*8%*)	8 (*14%*)	6 (*10%*)	40 (*68%*)	59		
**Monthly Income**	< 2000 yuan	25 (*10%*)	32 (*12%*)	30 (*11%*)	175 (*67%*)	262	3.46	0.749
	> 2000 – 6000 yuan	5 (*12%*)	6 (*14%*)	6 (*14%*)	26 (*60%*)	43		
	> 6000 – 10,000 + yuan	0	3 (*18%*)	1 (*6%*)	13 (*76%*)	17		

**signifies a significant result at the 95% confidence interval. ^a^signifies an adjusted residual that exceeded + 1.96, which indicates the number of cases in that crosstabulation cell was significantly larger than would be expected if the null hypothesis were true. ^b^signifies the number of cases was significantly lower than expected

### Students’ Use of Wildlife Products

**Fig. 1:**
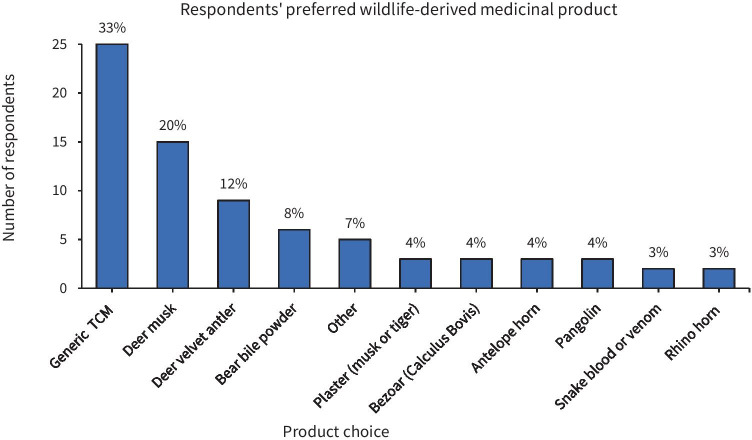
61% of respondents (91 individuals out of 149, with Non-Users instructed to skip this question) ranked animal – derived medicine as their most likely type of wildlife product to use or purchase. Figure 1 displays the stated medicinal product preference for respondents who chose medicinal products as their preferred type of wildlife product, when asked to give an example in an open-ended question. Note: the category ‘other’ includes Centipede (1 subject), Ambergris [sperm whale by-product] (1 subject), Deer blood (1 subject), “strong bone cream” (1 subject), and tiger bone wine (1 subject). TCM = Traditional Chinese Medicine

Three types of consumer behaviour were addressed in the survey – (1) using wildlife as food, (2) using medicine or tonic products containing wildlife-derived ingredients, and (3) ornamentation, wearing garments and/or owning curios made from wildlife-derived materials (Zhang *et al.,* 2008). Medicinal products emerged as the main use among respondents who had expressed past consumption and/or future interest in using wildlife products, with a mean rank ordering of preference of 1.39, 2.05 and 2.57 for medicine, ornamentation and food, respectively (1 equating to most likely to use, 3 to least likely). Where applicable, respondents were asked to specify a preferred wildlife product (Fig. 1). Deer musk was the most commonly mentioned medicinal wildlife product, followed by velvet antler and bear bile.

#### Motivational Drivers for Wildlife Product Use

Past and/or Future Users (i.e., respondents who had expressed using wildlife products, whether it be past — or intended future — usage) (*n* = 116) were presented with a series of sixteen statements (Table 2) and asked to indicate their level of agreement with each on a 5—point Likert scale where (1) was ‘strongly agree’ and (5) ‘strongly disagree’.

**Table 2:** Statements listed in the questionnaire pertaining to different wildlife product attributes

**Attribute type (predictor)**	**Statement**	Average score (1–5)	Standard Deviation
Medicinal /curative properties	‘I value wildlife products for their health benefits, curative effect to treat illness and promote wellness**.** Wild sourced products have greater medicinal strength’	2.7288	1.170
Tradition	‘A wildlife product that epitomises tradition and celebrates cultural heritage appeals to me’	2.8655	1.131
Authenticity	‘Wildlife products sourced from their natural environment are more desirable because they are of authentic origin, more potent or of greater purity’	2.9832	1.270
Rarity	‘I find wildlife products that are exclusive or highly sought-after desirable, even if they are harder to get hold of. For instance, products derived from an extinct — or rare, precious — species’	3.0084	1.348
Satisfying curiosity	‘On the whole, I find wild animal goods intriguing and desirable’	3.3025	1.172
Spirituality	‘Wildlife products with spiritual significance resonate most with me. I am drawn to items that can bring good fortune and protection to business or personal life’	3.4622	1.121
Collectible asset for investment	‘Wildlife products are a financial asset worth collecting for their market value’	3.5042	1.131
Nutrition	‘Wild-caught animal products are more nutritious and healthier than farmed products’	3.5882	1.119
Wild provenance	‘Imitations of wildlife products are an inferior choice to wild-caught’	3.6387	0.993
Prestige Food	‘When eating in a restaurant, I tend to order wild meat over farmed meat, even if it’s more expensive, because its more delicious or creates more of an impression amongst guests’	3.6975	1.025
Aesthetic	‘I value wildlife products for their aesthetic or decorative qualities. They can create a good Feng Shui and enhance the interior design’	3.7227	1.057
Gift	‘I would purchase wildlife products as gifts for valued business associates or close personal friends to show my respect and gratitude’	3.7542	1.077
High Price	‘A higher price of a wildlife product reflects the products greater value, superior quality or curative potency’	3.9576	1.021
Symbolic of Individuality	‘Purchasing prestigious wildlife goods is a way to express one’s individuality’	3.9916	0.918
Embodying virtues	‘I believe that consuming products derived from powerful animals can lead me to imbue such attributes, embodying their virtues’	4.1513	0.903
Symbolic of success	‘Using wildlife products symbolises wealth, sophistication or success, can enhance my image and reputation, and can also strengthen business exchanges and win the respect of others’	4.1681	0.861

Table **2** presents a summary of the descriptive statistics relating to the average Likert score (1 – 5) derived from each attribute statement. This series of statements was formulated to reflect different traits and values attributable to a wildlife product. These attributes are not mutually exclusive. The lower the average score, the greater the agreement with the corresponding statement and vice versa. This table gives an indication of the relative strength of different motivations and which attributes drive respondents’ choices. Displayed in ascending order from the wildlife product attributes that elicited the lowest score and therefore the greatest level of agreement, to the attributes that received the highest average score and therefore the lowest level of affinity

The sample’s mean response for each statement was analysed. The average scale scores ranged from 2.72 to 4.16. However, Table 2 shows that most of the average scores fall within 3 (equating to the response *Neither agree nor disagree*). A Kruskall Wallis analysis of variance test was conducted to examine the differences in the respondents’ level of affinity (score) depending on the type of attribute the wildlife product hypothetically exhibited. It revealed the distribution of participants' responses to significantly differ for at least one of the attributes *X*^*2*^_15_ = 235.875, *p* = < 0.001, therefore not all attributes were perceived to be of equal value. A post hoc multiple comparisons test revealed the difference in the degree of affinity orientation to lie between ‘medicinal properties’ (the only attribute with a median score of 2 which equates to the *agree* response); versus several lesser valued attributes possessing minimal appeal, indicated by respondents most frequently disagreeing with them. ‘Medical properties’ is therefore the attribute respondents assigned most value to; exemplified by 57.8% of participants either strongly agreeing or agreeing with the corresponding statement, with the lowest mean score of 2.72 (Table 2). Respondents were invited to make additional comments on what it is about wildlife products that resonates most with them (Appendix A). In the context of medicine, a reoccurring theme is summarised by participant NEFU342, explaining how they are “*not interested in wildlife products unless for some urgent thing, such as saving lives*”.

**Fig. 2:**
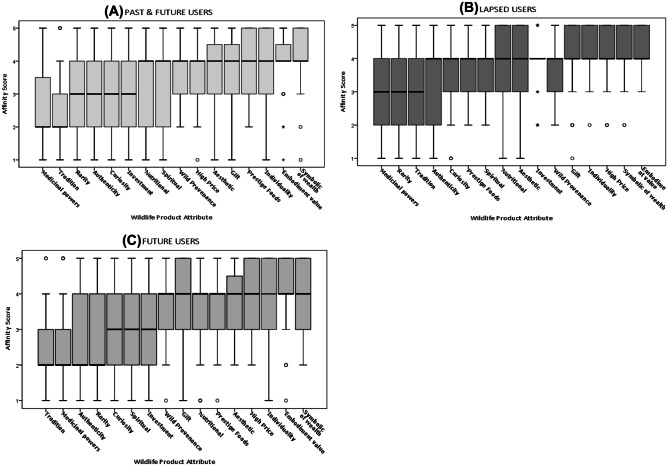
Boxplot charts depicting the degree to which respondents agreed or disagreed with 16 wildlife product attributes and the level of variability in response. Each chart displays the distribution of affinity scores for each consumer group (**A**) PAST & FUTURE USERS (**B**) LAPSED (FORMER) USERS and (**C**) FUTURE USERS across the categories of wildlife product attributes. For each consumer group, the x axis displays attributes in ascending order of affinity, from left – the attribute that elicited most agreement within the consumer group – to right – the attribute that received most disagreement by respondents within that consumer group. The y axis, labelled ‘affinity score’, spans from 1 (strongly agree) to 5 (strongly disagree). The thick horizontal lines indicate median values (a measure of central tendency), the boxes illustrate the interquartile range and the vertical lines indicates the maximum value or 1.5 times the height of the box and the ° represents a moderate outlier

**Fig. 3:**
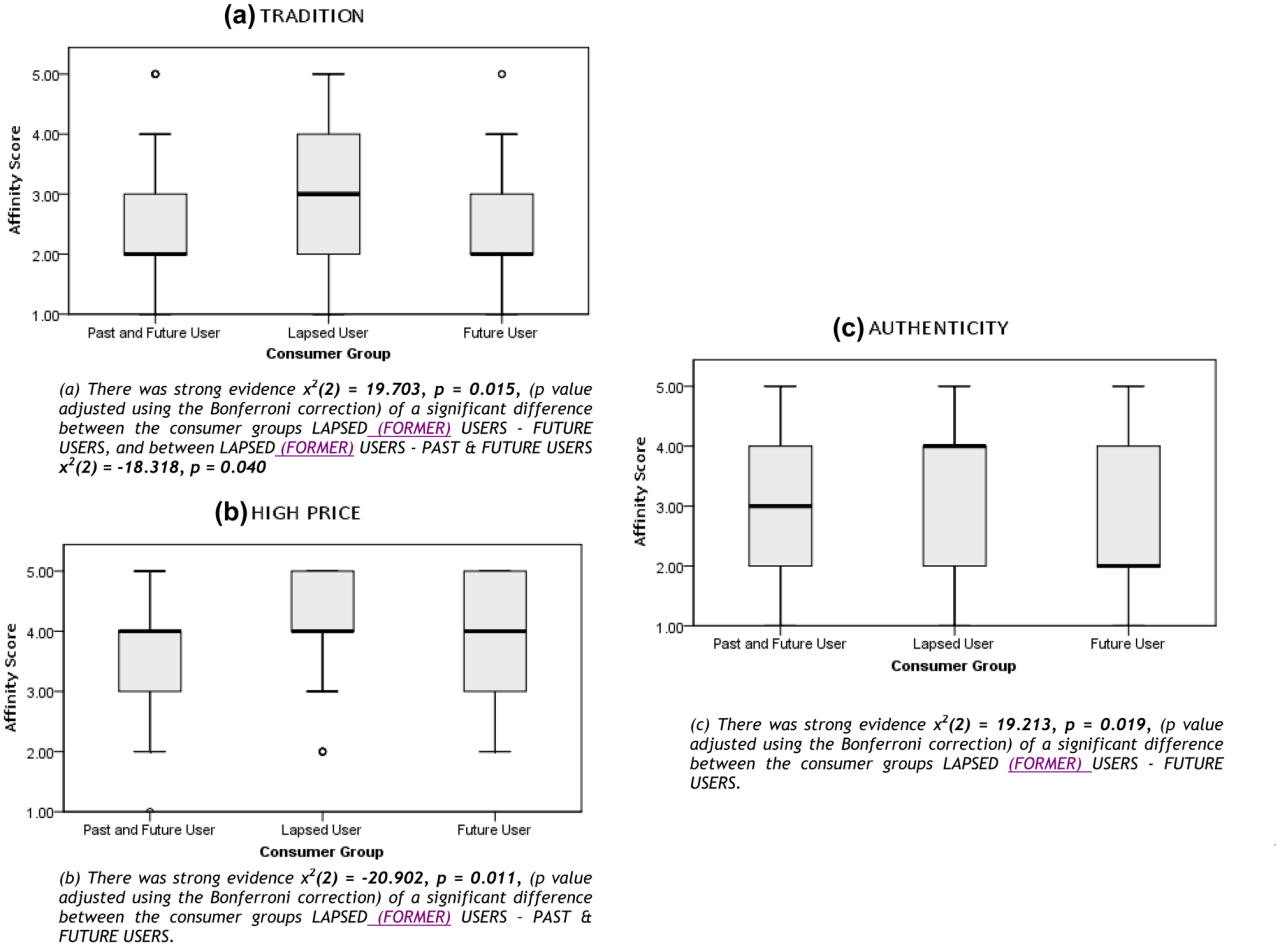
A series of boxplot outputs, displaying the significant results of an Independent Samples Kruskal Wallis test and a post hoc Dunn pairwise comparisons test, performed to compare all pairs of User Groups. A Dunn pairwise test revealed the distribution of respondent affinity scores were significantly different for the following attributes (**a**) TRADITION (χ2(2) =9.785, *p* = 0.008) (**b**) HIGH PRICE (χ2(2) =8.625, *p* = 0.013) (**c**) AUTHENTICITY (χ2(2) =7.505, *p* = 0.023), across the categories of consumer group, as featured on the x axis

Table 2 tells us that the attitudinal statements which elicited the greatest level of disagreement from respondents, and consequently the highest mean scores, were wildlife products being ‘symbolic of success’ (81.1% disagreed), conferring the ‘embodiment of virtues’ (82.8%) and an ‘expression of individuality’ (73.3%) respectively. These attributes also have the lowest standard deviation values (Table 2), indicative of a convergence in responses, such that there is more evidence of coherency in which attributes do not appeal to our sample, as oppose to which do. Second only to ‘medicinal properties’, ‘rarity’ elicited the highest proportion of respondents (49.2%) who strongly agreed or agreed this is a desirable attribute in a wildlife product. Yet, with both a mean of 3.03 and median value of 3 (equates to ‘*neither agree nor disagree*’ on the Likert scale) and the highest variability in responses (SD = 1.35), this infers a polarity of attitudes towards ‘rarity’. Similarly, despite a comparatively high level of agreement (45.7%) to ‘authenticity’ as a valued attribute in a given wildlife product, reflected in a mean score of 2.9, ‘authenticity’ possesses the second highest SD (1.27), implying heterogeneity in the distribution of responses to this attribute. Close to half of respondents (47.4%) agreed that wildlife products ‘epitomising tradition’ appealed to them, with a mean of 2.87. Notably, 78.4% disagreed that the higher the price of a wildlife product, the greater its value. The ‘Satisfying Curiosity’ statement elicited the greatest attitudinal ambivalence amongst respondents, with 25% neither agreeing nor disagreeing. A similar sentiment was summarised by respondent BUCT289 who explained how their “family have pangolin scales just because they are curious. I don't like killing and I have seen someone eat this animal but I never eat this. I like to watch videos about animals”*.* Breaking this attributed value to wildlife products down by User Group categories, Former Users were inclined to disagree more strenuously with ‘high price’ being a valued attribute, while Past & Future Users disagreed but to a lesser extent, in comparison to Future Users whose responses to ‘high price’ had the greatest within group variance, demonstrated by the boxes IQR spread (Fig. 3b). ‘Tradition’ was the attribute most valued by Future Users (Fig. 2C)*,* followed by ‘medicinal powers’, this order of preference summarised by ‘Future User’ respondent BUCM276 who stated “We shouldn't use [wildlife] except for urgent things, such as medicine and cultural inheritance”. Whereas ‘tradition’ was second only to ‘medicinal powers’ (first) as the attribute most valued by the Past & Future User group (Fig. 2A), and ‘tradition’ third only to the attributes ‘rarity’ (second) and ‘medicinal powers’ (first) for Former Users (Fig. 2B). Respondents’ affinity for the ‘authenticity’ of wildlife products was significantly different between Former Users and Future Users, both displaying equal IQR spread but Former Users’ dispersion of responses to ‘authenticity’ negatively skewed, with the median positioned at the upper quartile (equates to the *disagree* response) whereas the distribution of responses was positively skewed for Future Users, with a median closer to the lower quartile and therefore it’s data constitutes higher frequencies of lower value scores (equates to the *agree* response) (Fig. 3c).

#### Deterrents Dissuading Students from Using Wildlife Products

Respondents defined as Former Users (in other words those that had formerly consumed but do not intend to consume in the future), Future Users and Non – Users were asked to indicate their reasons for not presently using wildlife products. The survey revealed the most frequently cited reason for individuals not using wildlife products was concerns over the animal’s welfare (89%, *n* = 281 of responses stated this definitely or usually applies), and not believing wildlife products should be used in the first place (84%, *n* = 268), with females more likely to express such views. Males on the other hand appear more preoccupied by more practical concerns about availability and effectiveness (Table 3).

**Table 3:** Reasons for not using wildlife products which significantly differed between male and female respondents

*P* -value	Sex	Definitely applies	Usually applies	Don’t know	Rarely applies	Definitely doesn’t apply	Total
χ^2^(2) = 8.964, *p* = 0.011	‘Not worth using as the benefits are questionable’						308
	Male	43 26.9%	50 **31.3%**^a^	30 18.8%	30 18.8%	7 4.4%	160
	Female	30 20.3%	32 **21.6%**^b^	34 23.0%	41 **27.7%**^a^	11 7.4%	148
χ^2^(2) = 10.192, *p* = 0.006	‘Have attempted to use, but I was put off by the limited availability’						309
	Male	10 6.2%	24 14.8%	25 **15.4%**^a^	57 35.2%	46 **28.4%**^b^	162
	Female	3 2.0%	17 11.6%	10 **6.8%**^b^	55 37.4%	62 **42.2%**^a^	147
χ^2^(2) = 10.435, *p* = 0.005	‘Believe that wildlife products should not be used’						311
	Male	77 **47.5%**^b^	55 **34.0%**^a^	17 10.5%	12 7.4%	1 0.6%	162
	Female	101 **67.8%**^a^	27 **18.1%**^b^	8 5.4%	10 6.7%	3 2.0%	149
χ^2^(2) = 11.275, *p* = 0.004	‘Out of concern for animal welfare’						314
	Male	72 **44.4%**^b^	64 39.5%	11 6.8%	11 6.8%	4 **2.5%**^a^	162
	Female	92 **60.9%**^a^	48 31.8%	7 4.6%	4 2.6%	0 **0.0%**^b^	151

Table 3 displays the results from a Kruskal Wallis test where male and female respondents were significantly different in the extent to which they agreed with the following reasons as deterrents to using wildlife products χ^2^ = 12.5, (4 N = 313), *p* = 0.014. (1) ‘not worth using as benefits are questionable’ (overall 51% of respondents agreed, but males were more in favour of this reason than females) χ^2^(2) = 8.964, *p* = 0.011, (2) ‘have attempted to use but I was put off by their scarce availability’ (17% agreed, but females disagreed to this being applicable more strongly) χ^2^(2) = 10.192, *p* = 0.006, (3) ‘don’t believe wildlife products should be used’ ( 84% agreed overall, significantly more females strongly agreed than males) χ^2^(2) = 10.435, *p* = 0.005 and ‘animal welfare concerns’ (89% agreed, again females agree more strongly) χ^2^(2) = 11.275, *p* = 0.004

^**a**^adjusted residual z – score is significantly higher than expected

^**b**^adjusted residual z – score is significantly less than expected

Hygiene and health risks linked to zoonotic disease was an applicable deterrent to 70% (*n* = 221/ 316) of participants (again that is, Former Users, Future Users and Non-Users) as an influencer of decisions to not use wildlife products. Indeed, 96% (*n* = 335) of all individuals surveyed believed educating people about the risk of zoonotic disease transmission to be somewhat useful (65% useful, 31% some impact – Fig. 6). Factors such as feeling too ashamed to buy wildlife products (70%, *n* = 225/318) or using non-wildlife products with a similar function instead of wild-sourced products (66%, *n* = 208/315) also received a greater level of overall agreement from respondents. Two-thirds of respondents attributed their non – consumptive behaviour to being deterred by the risk of penalties (65%, *n* = 209/317) and possessing no interest or not having had the opportunity to consume wildlife (64%, *n* = 202/314). Furthermore, prohibitive cost was an applicable scenario for sixty percent (*n* = 190/316) of people, who were discouraged by the associated high prices as expressed by participant NEFU25 “university students do not have the ability to pay for the wildlife products”*.*

**Fig. 4:**
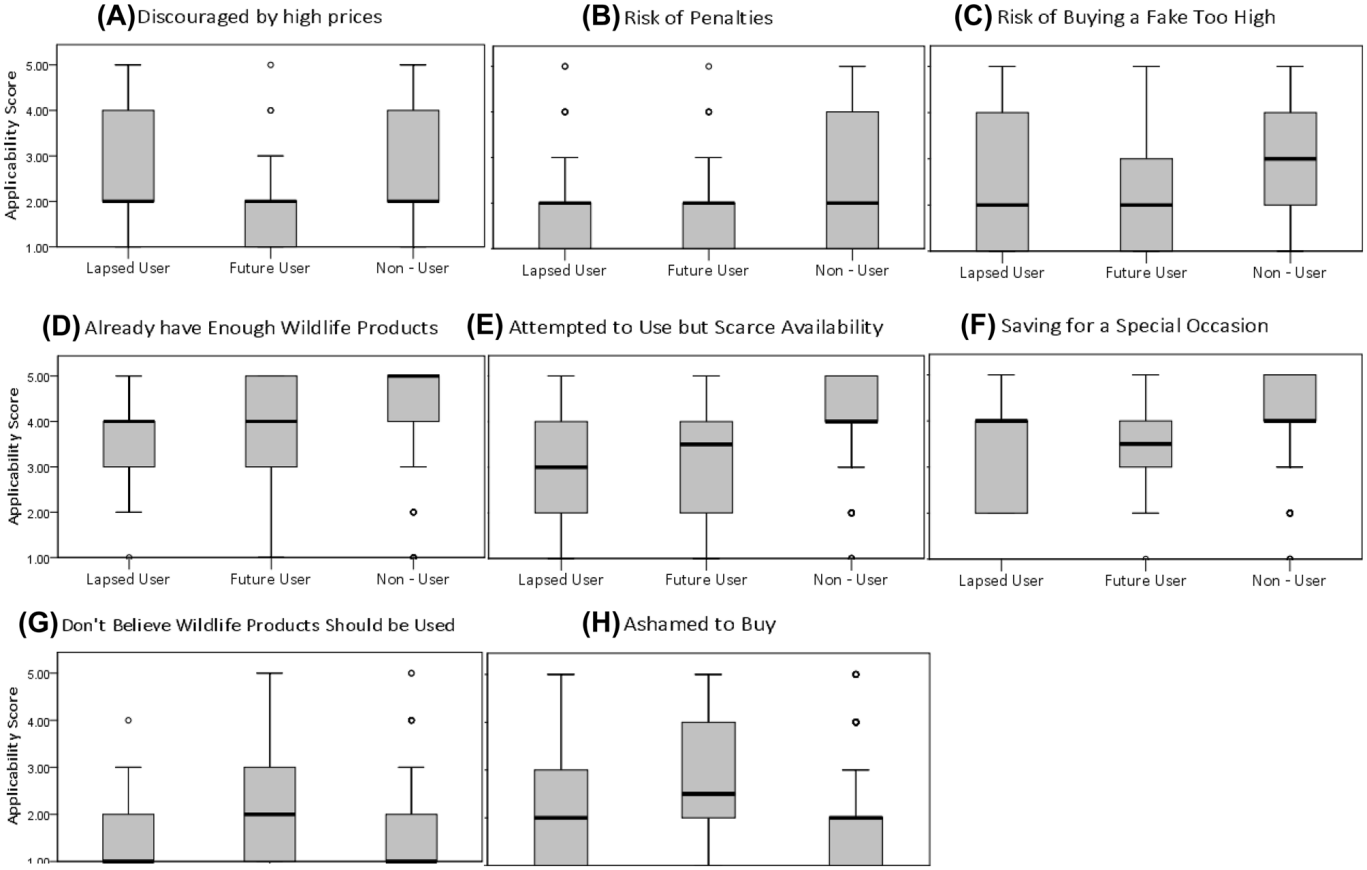
Boxplots visualising the significant results of a post hoc pairwise comparisons test. The following ‘reasons for non – consumption’ (**A**) - (**H**) significantly differed in their score between the different consumer groups, as seen along the X axis (Lapsed (hereafter ‘Former’), Future and Non - User), and applicability score features on the y axis, where 1 was ‘definitely applies’ and 5 was ‘definitely does not apply’. (**A**) High price χ^2^(2) = -54.874, *p* = 0.001 Future Users – Non – Users, (**B**) Penalties χ^2^(2) = -35.306, *p* = 0.044 Future Users – Non – Users, (**C**) Risk of Fakes χ^2^(2) = -59.598, *p* < 0.001 Future Users – Non – Users, (**D**) Enough Already χ^2^(2) = -47.554, *p* = 0.003 Former Users – Non – Users, (**E**) Scarce Availability χ^2^(2) = -58.759, *p* < 0.001, Former Users – Non – Users and χ^2^(2) = -56.715, *p* < 0.001 Future Users – Non – Users, (**F**) Special Occasion χ^2^(2) = -49.250, *p* = 0.002 Future Users – Non – Users and χ^2^(2) = -41.311, *p* = 0.017 , Former Users – Non – Users , (**G**) Against Beliefs χ^2^(2) = 68.341, *p* < 0.001 Future Users – Non – Users and χ^2^(2) = -53.508, *p* = 0.007 Former User – Future User, (**H**) Ashamed to Buy χ^2^(2) = -50.974, *p* = 0.025 Former Users – Future Users and χ^2^(2) = 47.018, *p* = 0.003 Non – Users – Future Users

Dividing these inhibitors for the consumption of wildlife products by User Group (see Fig. 4), ‘Discouraged by High Prices’ was an applicable deterrent from consumption for a significantly greater proportion of Future Users (displaying lower within group variance, Fig. 4A), compared to that of Former and Non-Users. Correspondingly, Non-Users displayed significantly different applicability scores in response to ‘Risk of Penalties’ being a reason stopping them from using wildlife products, compared to Future Users, with the latter’s dispersion of responses more homogenous, indicated by the boxes IQR spread (Fig. 4B). Likewise, ‘Risk of Buying a Fake too High’ as a motivation behind students choosing not to use wildlife products was an applicable scenario for a significantly greater proportion of Future Users, indicated by a lower mean rank of 100 compared to Non-User’s mean rank of 160 (Fig. 4C). ‘Don’t Believe Wildlife Products Should be Used’ elicited a significantly greater frequency of lower value scores (signifying greater applicability) from Non-Users (mean rank = 139), whose median value is positioned at the lower quartile range equating to 1 – *definitely applies,* as well as Former Users (mean rank = 153), compared to Future Users (mean rank = 207) whose distribution of responses displays greater polarity towards this reason for non-consumption (Fig. 4G). This same trend of Future Users agreeing less strenuously is echoed in the significantly differing responses elicited by ‘Ashamed to Buy’ (Fig. 4H) between Former/Non-Users (both displaying a median value equating to the ‘*usually applies*’ applicability score and with a mean rank of 140 and 143 respectively) compared to Future Users possessing a mean rank of 191, indicating a higher frequency of higher value scores (signifying lower levels of applicability).

#### Specific Perspectives on Wildlife Consumption Policy

**Fig. 5:**
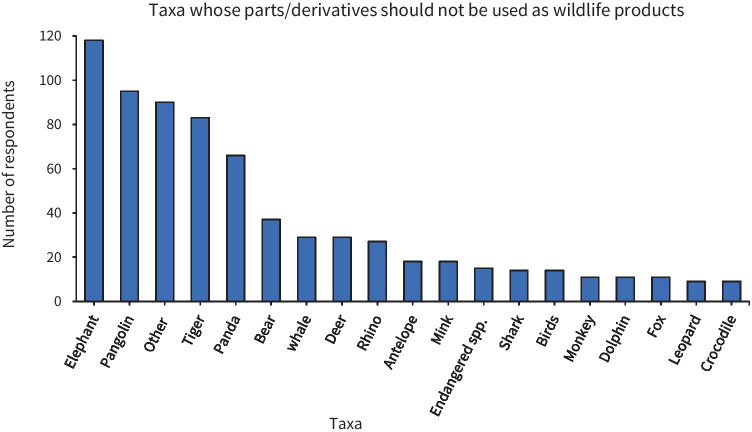
Taxa most frequently cited by respondents as not appropriate for use by humans as wildlife products. *Where taxa was cited to the taxonomic classification of species level, the taxonomic nomenclature was listed below. Note the category ‘Other’ includes: Cat (8 subjects), Cheetah (1 subject), Chinese alligator *Alligator sinensis* (CITES App. I/EU Annex A) (2 subjects), Chinese sturgeon *Acipenser sinensis* (App. II/Annex B) (7 subjects), Coral (1 subject), Dog (7 subjects), Duck (1 subject), Elk (5 subjects), Frog (3 subjects), Caterpillar Fungus *Ophiocordyceps sinensis*] (1 subject), Giant salamander (4 subjects), Giraffe (3 subjects), Hare (1 subject), Kangaroo (1 subject), Koala (2 subjects), Lion (4 subjects), Moose (1 subject), Pigeon (2 subjects), Rabbit (4 subjects), Raccoon (1 subject), Red crowned crane *Grus japonensis* (App. I/Annex A) (6 subjects), Scorpion (1 subject), Seal (3 subjects), Snake (7 subjects), Squirrel (1 subject), Toad (1 subject), Turtle (1 subject), Wild fish (1 subject), Wild pig (2 subjects), Wild yak (2 subjects), Wolf (6 subjects). This was an open-ended question. Therefore, respondents could list multiple species whom they believed their bodies/parts/derivates shouldn’t be used as wildlife product(s), hence the cumulative total may exceed the total sample size

All respondents (that is, Past & Future Users, Former Users, Future Users and Non-Users) were asked to recall if they had a species* in mind that they believe should not be used as a wildlife product (Fig. 5).

**Fig. 6:**
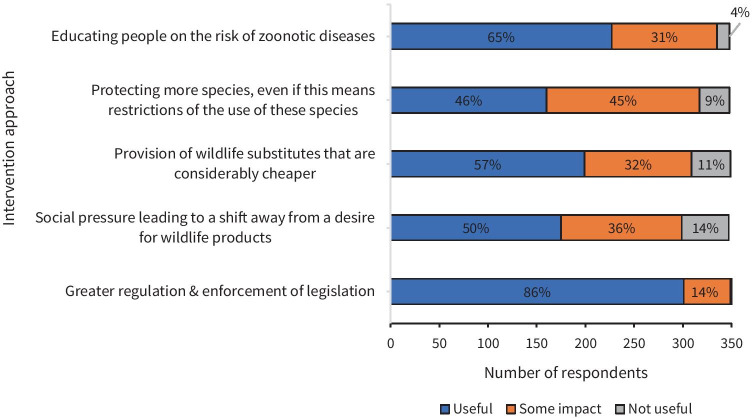
Bar graph visualising the degree to which respondents perceive different intervention strategies to be useful in mitigating unsustainable wildlife harvesting

The majority of survey participants (89%, *n* = 311/341) strongly agreed or agreed that wildlife consumption should not be allowed if the animal is protected and/or threatened by extinction (Table 4). Correspondingly, ‘greater regulation and enforcement of legislation’ was unanimously considered to be the most effective strategy to mitigate unsustainable wildlife consumption amongst respondents (Fig. 6). Social pressure that leads to a shift away from the desire to use wildlife products received the highest proportion of respondents viewing this measure as not useful in reducing over-harvesting of wildlife (14%) (Fig. 6). Fifty-seven percent of respondents said providing wildlife substitutes that are considerably cheaper would be a useful strategy for mitigating unsustainable wildlife harvesting, 32% said it would have some impact while 11% thought it would not be useful (Fig. 6).

**Table 4:** Statements listed in the questionnaire pertaining to the circumstances under which wildlife consumption should or should not be allowed

**Wildlife Consumption Attitudes**	% agreement **	N *	Average Score (1 -5)	Standard Deviation	Viewpoint category
‘Not if the animal is threatened by extinction or protected’	88.8%	311	1.5837	0.849	Conditional utilisation (species status dependant)
‘No, wildlife should never be used for human benefit, species have an intrinsic right to co-exist with humans’	69.7%	244	2.0439	1.122	Biocentric (explicitly opposed due to intrinsic values)
‘Yes, but only if the given wild animal product has a legitimate and proven benefit’	71.4%	250	2.4075	1.131	Conditional utilisation (utilitarian values)
‘Yes, but only in the case of an emergency e.g., poor health, as a last resort’	59.4%	208	2.6880	1.235	Conditional utilisation (emergency)
‘Yes, but only for wild animals that are plentiful’	48.5%	170	2.8463	1.211	Conditional utilisation (species status determined)
‘I don’t know enough about this subject to confidently answer this question’	25.2%	88	3.1515	1.081	Undecided
‘Yes, humans have a right to use animals as a natural resource for our own benefit’	16.9%	59	3.8294	1.110	Anthropocentric
‘I am indifferent either way’	7.2%	25	3.92281	1.007	Indifferent
‘Yes, but only sparingly for special occasions like New Year or weddings’	8.3%	29	3.94444	0.906	Conditional utilisation (occasional)

Table 4 presents the average Likert score in response to each attitudinal statement, a score of 1 representing ‘strong agree’ and 5 ‘strongly disagree’. Statements were designed with a shifting focus from anthropocentric outlooks, to context-specific scenarios, to biocentric viewpoints, as seen in Zhang and Yin’s (2014) study which categorises peoples’ attitudes towards wildlife consumption into cognitive types. Displayed in ascending order, from viewpoints that elicited most agreement to most disagreement

*As respondents were instructed to score each wildlife consumption viewpoint, the sum of the total percentages exceeds 100%

**% signifies the proportion of respondents who strongly agreed or agreed with each viewpoint on wildlife consumption

The anthropocentric outlook that ‘humans have a right to use animals as a natural resource for our own benefit’ elicited a low level of agreement (17%), whilst over 76% of respondents strongly disagreed or disagreed with this viewpoint (*n* = 262/346) (Table 4). On the other hand, 72% (*n* = 250/346) of the total individuals surveyed strongly agreed or agreed that wildlife consumption should be allowed only under the circumstances whereby the given product has a legitimate and proven benefit. Yet 71% of respondents (*n* = 244/341) adopted a more biocentric outlook, believing wildlife should never be used for human benefit since they have an intrinsic right to coexist with humans.

### Attitudes to Substitutes

On average neither sex believed substitutes were a poor choice compared to wild-harvested, with 75% of responses constituting strongly disagree or disagree, although males were more likely to agree than females (Table 5).

**Table 5:** Male and female respondents responses to the statement 'Imitations of wildlife products are an inferior choice to wild-caught’

**Sex**	Strongly agree	Agree	Neither agree nor disagree	Disagree	Strongly disagree	Total *176*
Male	2 2.0%	13 12.7%	30 29.4%	45 44.1%	12 **11.8%**^**b**^	102
Female	0 0%	6 8.1%	12 16.2%	34 45.9	22 **29.7%**^**a**^	74

Table 5 presents the results of a Kruskal Wallis test which revealed the level of agreement to the attitudinal statement ‘imitations of wildlife products are an inferior choice compared to wild-caught’ to differ significantly between sexes (*χ*^2^ 1 4.042, *p* = 0.04). Female respondents typically reacted more strongly against this statement than males, indicated by a lower mean rank of 54.13 for males (between rank 3 – 4 on the Likert scale) versus 66.26 for females (4 -5)

^**a**^adjusted residual z – score is significantly higher than expected

^**b**^adjusted residual z – score is significantly less than expected

**Fig. 7:**
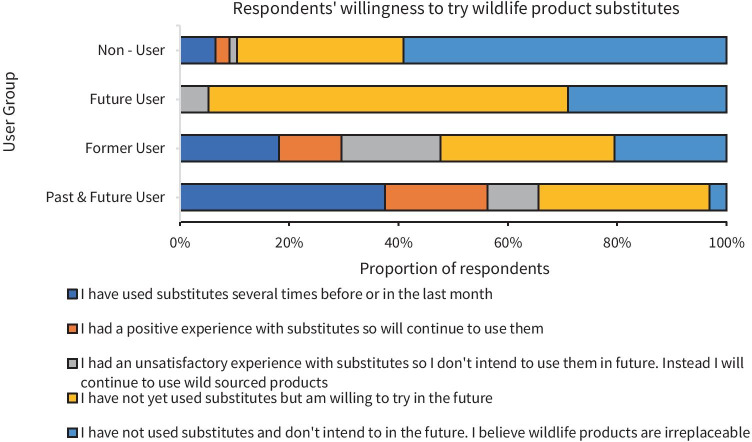
A Kruskal Wallis test revealed there to be a significant difference between the response variable – willingness to try substitutes and the explanatory variable – affinity for wildlife products χ^2^ 3 76.453, *p* = < 0.001, (the latter categorised into Lapsed (Former) Users, Past & Future Users, Future Users and Non – User consumer profiles)

An overall mean of twenty percent (*n* = 68) of total respondents had tried a wildlife substitute before. Breaking this down by User Group (a measure of respondents’ affinity for wildlife products), 70% of Past & Future Users: (*n* = 21/32), 48% of Former Users: (*n* = 21/44), 10% of Non – Users: (*n* = 24/230) and only 5% of Future Users: (*n* = 2/38) had tried a wildlife substitute prior to the survey (Fig. 7). Within this group of consumers who had previously tried substitutes, a strong majority of 76.5% (*n* = 52 of 68) intended to continue to use substitutes based on their past experience(s). These experiences with product substitution that shaped respondents’ inclination to continue using substitutes constituted 56% having used a synthetic substitute, followed by 25% a farmed, non-domesticated animal substitute, 15% a domesticated animal alternative and 6% a different wildlife species from the one they’d typically opt for; the latter type of substitution was exclusive to the Past & Future User group (Table 6).

**Table 6:** Examples of substitutes tried by respondents who have used substitutes and intend to continue using (*n* = 52)

**Substitute type**	User Group categories (affinity for wildlife products)				Total (row)	% Total
	**Past & Future User**	**Former User**	**Future User**	**Non-User**		
**FARMED**	4 (*22% of Past & Future Users who tried substitutes*)	5 (*33% of Former Users who tried substitutes*)	0	3 (*16% of Non-Users who tries susbtitutes*)	12	23.1%
*Cited farmed substitutes*	Chinese sturgeon (1), Aquatic product (1), Animal fur (1)	Native chicken (1), Farmed ivory (1)	N/A	Leather (2), Buffalo horn (1)		
**DIFFERENT WILDLIFE SPECIES**	3 (*17%*)	0	0	0	3	5.8%
*Cited wildlife substitutes*	Ox horn (1), Buffalo horn (1)	N/A	N/A	N/A		
**SYNTHETIC**	6 (*33%*)	8 (*53%*)	0	15 (*78%*)	29	55.8%
*Cited synthetic substitutes*	Artificial deer musk (1), Artificial fur (1), Artificial leather (1), Synthetic Medicine (1)	Artificial leather (2), Ointment (1), Artificial fur (1), Plush toy (1), Artificial *calculus bovis* (1), Synthetic Mink (1)		Artificial leather (7), Artificial *calculus bovis* (2) Artificial fur (2), Synthetic down (feather) jacket (1), Artificial milk product (1), Medicine (1), Synthetic Pearl (1), Synthetic Wool (1)		
**DOMESTICATED**	5 (*28%*)	2 (*13%*)	0	1 (*5%*)	8	15.4%
*Cited domesticated substitutes*	Farmed giant salamander (1), Domestic chicken (1), Bamboo rat (1), Common leopard gecko (1)	Wooden furniture (1)		Pork/beef (1)		
Total (column)	18	15	0	19	52	

Table 6 displays the positive substitution experiences of those respondents who stated they had used wildlife product substitutes before and intended to continue using wildlife product substitutes (*n* = 52). This table lists the wildlife product substitutes cited by respondents when asked to recall the name and type of substitute they’d used most recently

Within the remaining 80% of total respondents (*n* = 276) who had not yet used wildlife substitutes, 43% stated their willingness to try them in the future (*n* = 119/276). Just over half of those individuals who had not tried substitutes but were willing to in future alluded to wanting to try a synthetic substitute (namely, false shark fin, artificial leather, faux fur, and synthetic wool) whilst a quarter were interested in using a farmed wildlife product (i.e., farmed deer, farmed fur).

In summary, amongst all respondents, irrespective of their User Group (i.e., affinity for wildlife products), 49.7% indicated that they were willing to use substitutes (whether it be a continuation of use or trying for the first time) for wildlife products in the future. This opinion was more of a majority (87.5%) among the ‘Past & Future’ User Group. Likewise, ‘Future Users’ possessed the greatest propensity to try substitutes for the first time (66% willing) (Fig. 7), whilst Non – Users of wildlife products (*n* = 230) were the least willing to use substitutes (only 39.6% would accept substitutes). Lastly, 50.3% of total respondents stated their intentions to not use wildlife substitutes in the future (comprised of 45.4% of total respondents who did not intend to try substitutes since they believed wildlife products to be irreplaceable and a further 4.6% who instead, intended on continuing to use wild derived products due to an unsatisfactory experience with wildlife substitutes).

Economic reasons were the dominant reason expressed by respondents’ for using a substitute because it was cheaper (34.5%) or because wild products were too expensive (7.5%). Conservation of wild populations and ecosystem were cited by approximately 20% of respondents (Table 7).

**Table 7:** Reasons given by consumers of substitutes as to why they used a substitute instead of a wildlife product

Themes for choosing substitutes instead of wildlife products	Number of respondents	Frequency %
Substitutes are cheaper	105	34.5
To conserve wildlife/ reduce extinction of wild populations and balance ecosystems	60	19.7
Substitutes are of equal quality as wildlife products/meet human needs	23	7.6
Wildlife products are too expensive	23	7.6
Wildlife product are not necessary/ I have no desire for them	13	4.3
Wildlife is protected by law; substitutes are legal	13	4.3
Substitutes are more readily available/ accessible, convenient, have sufficient resources	11	3.6
Wildlife products have limited availability	11	3.6
Wildlife are our equals, therefore have the right to co-exist with humans	11	3.6
Substitutes are safer and/or healthier	8	2.6
Substitutes perform better/ I prefer them/ more suited to my needs	6	2.0
Wildlife carry diseases/ are unhygienic/ not safe to consume	6	2.0
More ethical to use substitutes, cruel to use wildlife products	6	2.0
Wildlife shouldn’t be killed to meet people’s desires	4	1.3
I experience substitutes in order to spread the word and replace wildlife products	3	1.0
Substitute recommended to them	1	0.3

Table 7 respondents were asked to express their reason(s) for using a substitute as opposed to a wildlife derived product. Respondents were able to offer multiple responses; hence the cumulative total may exceed 100%

Generally, a low level of familiarity with substitutes for wildlife products existed amongst our sampling frame, with several individuals anecdotally noting that the questionnaire was the first they had heard of wildlife product substitutes. However, of those that were aware of substitution, respondent’s education was found to be the greatest influencer in introducing participants to wildlife substitutes, with one third selecting this as the most influential factor in how they heard about them. Forty-five percent of respondents reported the internet and social media as their first or second most important source of information in familiarising them with substitutes, rendering this the second biggest influencer. Thirdly, television/radio advertisements closely followed in influence.

#### The Propensity to Try Specific Substitutes

An assessment of substitutability was conducted, with a range of candidate substitutes for wildlife commodities (each raised in the wider literature as potential substitutes for their wildlife product counterparts) presented to the respondents to choose their preferred wildlife product for substitution. This assessment works on the assumption that participants will choose the product that provides them with the highest level of utility (Thomas-Walter *et al.,* 2020). Of the surveyed 350 participants, 65% (*n* = 228) selected fur as the wildlife product they were most likely to substitute (out of a choice of four different wildlife products), with just over half of these 228 individuals having worn or owned real animal fur before. Twenty three percent of total individuals (*n* = 80/350) selected Deer Musk as their most substitutable wildlife product, with significantly fewer (21%, *n* = 17/80) having already tried this product before. Fifty-nine percent of the 76 individuals who selected Abalone as their most likely product to substitute (22% of total respondents) had consumed this product before, whilst only 24% of the 68 individuals who chose Velvet Deer Antler (19% of total respondents) had used it before.

**Fig. 8:**
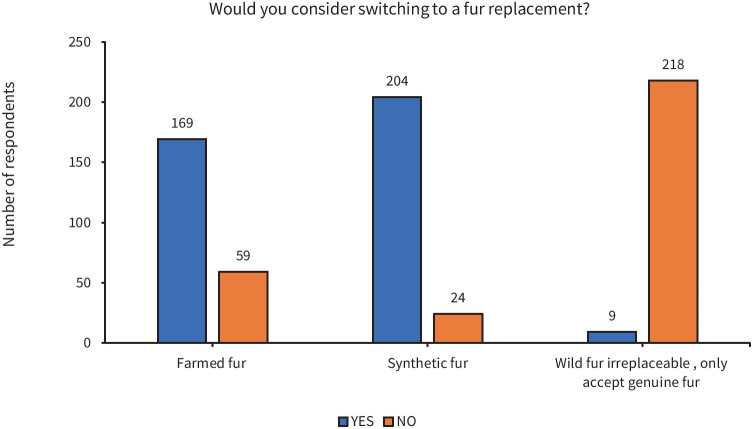
The proportion of respondents who would consider trying different fur substitutes

The types of substitutes that students could choose were based on those types of ‘alternatives’ outlined in Broad & Burgess study (2016). Due to the different consumptive patterns connected to the four candidate wildlife products (fur, deer musk, abalone and velvet deer antler) and the complexities related to what substitutes have been identified for their wildlife product counterpart, the range of substitutes — on the market and therefore — available for respondents to select varied. Differential selection frequencies were observed for different substitute alternatives: the highest being *synthetic* fur, with 89% (*n* = 204/228) stating they would switch to this fur replacement (Fig. 8), compared to 83% for *synthetic* deer musk (*n* = 66/80). Comparatively, switching to a *farmed* fur substitute was accepted by three-quarters of the 228 participants who selected fur, whilst only 4% (*n* = 9) believed wild animal fur to be irreplaceable, therefore only settling for the genuine article. In comparison to *farmed* fur product substitution, a greater proportion (86%, *n* = 65/76) were willing to switch to *farmed* Abalone and *farmed* Velvet Antler product (85%), yet less were this way inclined when it came to *farmed* Deer Musk (73%). The *plant* substitute Acorus rhizome (*Acorus tatarinowii)* for Deer Musk achieved a high 86.8% acceptance rate whilst Ginseng as a *plant* substitute for Velvet Antler received a slightly lower 78% acceptance. Abalone *plant* substitute Konjac (*Amorphophallus konjac*) elicited a 67% acceptance rate whilst a higher proportion of respondents (80%) were willing to try a less scarce seafood product as an alternative to Abalone.

A larger portion of respondents disagreed (44%), than they did agree (36%) with the statement ’musk is only authentic if cultivated from wild animal species, but it does not necessarily have to be derived from the musk deer’ whilst 20% neither agreed nor disagreed with this attitudinal statement, signifying less of a consensus amongst respondents’ viewpoints – Specifically, 69% of respondents who selected Deer Musk as their most substitutable wildlife product were prepared to try Muskrat, Ox or Civet as an alternative wildlife species substitute for Deer Musk. The lowest propensity for a substitute was *synthetic* Velvet Antler, with 63% willing to try this replacement, with the Deer Musk alternative Ambergris (Lóng xián xiang) — a waste product sourced from the Sperm Whale *Physeter macrocephalus* (App. I/Annex A) — not far behind it (63.8%). In contrast, ‘I would try to use Ambergris as it’s harvest does not harm the wildlife species’ received the highest level of agreement elicited out of a series of attitudinal statements posed to those respondents who selected Deer Musk (see Appendix B), with 68% strongly agreeing (17%) or agreeing (51%), only 18% disagreeing and 14% neither agreeing nor disagreeing.

## Discussion

### The Prevalence of Consumption

Consumptive wildlife use is deeply entrenched within China’s heritage and societal norms, with utilitarian points of view towards wildlife historically widespread in Chinese culture (Swan & Conrad, 2015; Thomas-Walters *et al.,* 2020), such that the prevailing mindset traditionally regards wildlife as an open resource to be exploited for human benefit, with economic returns often emphasised (Zhang & Yin, 2014). Having said this, the importance of biodiversity conservation is increasingly recognised in China (Coals *et al.,* 2020; Zhang & Yin, 2014), partly exemplified by the closure of domestic elephant ivory markets in China at the end of 2017 (Zhou *et al.,* 2018) as well as revisions made to the ancient Traditional Chinese Medicine (TCM) Pharmacopoeia of the People’s Republic of China in an attempt to reduce the use of tiger (*Panthera tigris)* (App. I/Annex A) bone and rhinoceros (*Rhinocerotidae*) (App. I/Annex A) horn as highly sought-after medicinal ingredients (TRAFFIC, 1997). And, more recently still, the up-listing of China’s three native pangolin species – Chinese (*Manis pentadactyla*), Sunda (*Manis javanica*), and Indian (*Manis crassicaudata*) (all App. I/Annex A) to First Class Special State Protection under the Wildlife Protection Law, the highest form of protection under national legislation, and subsequent removal of pangolin scales (Squama Manitis) as a listed raw ingredient in the Pharmacopeia 2020 edition (Jin *et al.,* 2020; State Forestry & Grassland Bureau, 2020). Indeed, Fabinyi and Liu (2014) and Fabinyi (2012) highlighted how such consumption is not necessarily an unalterable tradition, but rather a historically based social practice that is subject to change. Our survey demonstrates that this sample of the student population exhibits a low overall affinity for wildlife products, with over three-quarters of those surveyed not reportedly using any wildlife products yet and crucially only a minority of those that had not used wildlife products expressed an interest in potential future usage (11%). Upon closer inspection, little heterogeneity existed within respondents’ affinity for wildlife products across demographic variables (Appendix A), suggestive of an emerging consensus in the form of these students’ low usage intentions towards wildlife goods. Therefore, we can infer that demand in this group is not currently prevalent, this survey finding little evidence to suggest a conspicuous surge in demand for wildlife products from China’s millennials would be likely to materialise, but rather, grounds for a possible diminishing use of wildlife products in this demographic. This finding conflicts with our initial assumptions, based on earlier research (IFAW, 1998; Meijer *et al.,* 2017; Wasser & Jiao, 2010; Zhang *et al.,* 2008; Zhang & Yin, 2014) which predicted young people’s potential high consumption and studies such as Chen *et al.* (2007) who found the use of Traditional Chinese Medicine in Taiwan to peak in those in their 30’s followed by 20’s; suggesting young adults were the prime consumptive age, in the past. Our study challenges perceptions concerning Chinese millennials’ driving the growth of demand for wildlife products, some of which were formed well over a decade ago. Consequently, this thinking may be outdated and no longer the case, especially amongst the new generation, but also in the context of Harbin, a decade having passed since the last similar research was undertaken in this locality (Wasser & Jiao, 2010).

There are numerous defining cultural and historical factors that may have shaped a shift in attitudes towards consumption of wildlife derived products in China’s contemporary society towards more biocentric and egalitarian values framed around animal welfare and conservation consciousness. Such contributing factors include Chinese millennials being the first generation to grow up entirely in a reformed era, where societal conditions and market liberalisation, is shaping a distinctive world view (Moore & Chang, 2014; Wang & Xu, 2009). For instance, university students are thought to be more exposed to foreign ideologies and a comparatively more open-minded and individualistic generation in relation to their elders (Davey, 2006; Moore, 2005). Indeed, China’s educational attainment has improved, with the nation’s 1979 to 2015 one-child-policy thought to have encouraged families to channel their savings into their only child’s education (Moore, 2005), with China’s gross enrolment ratio into tertiary education having risen year on year from 24.2% of 18–22 years olds enrolled at university in 2010 compared to 50.6% by 2018 (UNESCO Institute for Statistics, 2019). According to Zhang and Wang (2011)**,** Chinese university students have received a systematic higher education and represent opinion leaders among young people, likely to form the core of young, aspiring elites with high disposable incomes.

### Context of — and Values Driving — Consumption

A high level of overall disagreement towards wildlife products’ given attributes being of any merit was captured in the survey, with 57% the highest level of agreement prompted by any attribute (medicinal powers); compared to 89% being the highest rate of applicability elicited by any factor dissuading respondents from using wildlife products (animal welfare concerns). Although only a small proportion, even so, a degree of interest was expressed for wildlife products amongst survey participants, with medicine consistently the most widely preferred, both as a type of consumer behaviour — using medicine or tonic products containing wildlife-derived ingredients, superior to ornamental curios and wild meat —as well as its perceived curative values. This result highlights the salience of medicinal properties as a correlate for wildlife product use, although vestigial, amongst these students. Accordingly, Wasser and Jiao’s (2010) report found those with higher education were significantly more likely to access Traditional Chinese Medicine than those without. Conversely, Liu *et al.* (2017) found surveyed college students in Beijing (mainly young citizens with a higher education) were significantly less willing to use bear bile medicine than older Beijing citizens and those with lower education levels.

Medicinal health benefits are intrinsically utilitarian by nature, fulfilling a functional purpose (Dang Vu & Nielsen, 2018). Correspondingly, wildlife products possessing a legitimate and proven benefit were strongly valued amongst our respondents. What’s more, our survey respondents emphasised the worth of wildlife medicines more in the context of a serious ailment or emergency event. This brings into question whether young people’s consumptive habits are likely to change as they age and become more reliant on regular medication. Indeed, Drury (2009) found age to be positively correlated with the use of wild animal-derived medicinal products in Hanoi, Vietnam.

When interpreting the study’s finding – i.e., that curative value prevailed as the main determinant of wildlife consumption by a considerable margin – it is worth noting that almost a quarter of survey respondents were enrolled at a university of Chinese medicine. It would be reasonable to surmise that, due to their training in Chinese medicine, which traditionally uses wild harvested plants — and animal — derived ingredients, this population of the sampling frame may have a different understanding of the use of wildlife compared to the rest of respondents. This was considered in the study design, such that one medicine university was sampled in each city to increase the validity of comparisons made between locations, and stratification of the sample was also applied to cover a range of medicine-related disciplines (including clinical medicine, traditional Chinese pharmacology, oncology, health management, acupuncture and massage, and pharmaceutical engineering). Nonetheless, this spread of respondents could have artificially amplified “curative value” as the most important characteristic.

The next most favoured attributes of wildlife products given in order of frequency were Rarity, Tradition, Authenticity and Gift Suitability. All such features have connotations with both hedonic and utilitarian desires (Dang Vu & Nielsen, 2018), whereby the impetus to use wildlife products is grounded in emotional and functional values, respectively. For instance, rarity — conferring status, symbolism of wealth and pride of ownership (Shairp *et al.,* 2016). Thus, will demand increase as this group ages and become more aware of the perceived benefits to some in terms of social esteem and career advancement that access to rare and exotic wildlife products could bring, or are respondents’ low affinity anticipated to persist. The majority of our surveyed students (81.3%) belonged to the lowest pay bracket, earning < 2000 yuan monthly (Appendix A), a foreseeable outcome considering their full-time commitment to higher education. So, as with the usage of wildlife medicine rising as individuals age, we must contemplate whether this current low attraction to wildlife goods would differ if their standard of living and disposable income increased, as it is likely to do so after they graduate and establish themselves into a career.

### Concerns Associated with Wildlife Products

Although multifaceted and not attributable to a single determinant, this survey reveals that the motives for avoiding wildlife products largely concerned the impact of consumption on animal welfare (female students to a significantly greater degree believers in pure protection and egalitarian values than males), along with environmental degradation, and hygiene-related reservations. Picking up on the latter motive, our research revealed a high level of awareness amongst students pertaining to the link between zoonotic disease transmission and other health problems associated with wildlife consumption, evidenced by 70% considering this explanation applicable to why they’re not currently consuming wildlife products; as well as deeming educating people on the threat zoonotic disease spill-over poses to human beings’ health one of the most effective measures for mitigating unsustainable wildlife use. Indeed, it has been noted in the academic literature that food safety is one of the most crucial issues for Chinese consumers (Fabinyi & Liu, 2014). Whereas, Ho and Shea's (2016) report revealed environmental concerns to be the most popular reason why respondents ceased consumption of shark fin soup (43.7%), and to a significantly lesser extent, shark fin soup’s cost (24.6%). Correspondingly, Wasser and Jiao (2010) postulates ‘hazards to health’, and ‘deterioration of the natural environment’ as approaches to campaign messages that could catalyse more immediate behavioural change. In all likelihood, these expressed zoonotic disease and health-related concerns in connection with wildlife product consumption are substantially heightened by the COVID-19 virus (designated as SARS-CoV-2) suspected origin in a wildlife market, bringing the potential implications of demand for wildlife products and substitutes alike to the fore.

The nature of our sample’s motivators and inhibitors is a telling finding that substantiates our respondents’ reported low prevalence of consumption; on the grounds that their reasoning stems more from personal principles, and a genuine internalisation of these opinions, as opposed to being founded solely on a respondent’s current financial situation or lack of accessibility, these being more temporary barriers they wish to overcome. Or likewise, by virtue of fear of detection and repercussions for disobeying regulations that they have no connection with (Swan & Conrad, 2015). For these reasons, this demographic’s motives for avoiding wildlife product consumption are more likely to manifest into enduring non-usage, thus complementing rather than undermining protective legislation (Swan & Conrad, 2015). In contrast to our research, limited availability in the market, illegality, and expensiveness were identified as the three key barriers to wildlife consumption in Wasser and Jiao (2010) study of six Chinese cities, as well as being contrary to the findings of Drury (2011a) study in Vietnam, where money was identified as the main factor preventing individuals from consuming wildmeat.

### Attitudes on Substitution

One of the major findings of significance in this survey was a potentially captive audience for substitutes present in this student demographic, indicated by half of the participants expressing a willingness to try substitutes. More encouragingly still, despite close to half of Past and/or Future wildlife product users believing authenticity to be a valuable trait, most respondents did not view wildlife product imitations as an inferior choice to wild-harvested goods. What this tells us is that these northern Chinese students don’t appear to exhibit a strong preference for wild provenance, rather they are more concerned about not compromising on quality so the issue of fakery is perhaps more of an inhibitor of consumption. According to Beijing seafood restaurant stakeholders in Fabinyi & Liu’s study (2014), it was the presence of ‘fake’ shark fin circulating in the market, this fakery associated with poor quality and health problems e.g., perceived toxicity of counterfeit fins, that acted as a greater driver to opt for a shark fin alternative, more so than the notion that their consumption is contributing to wild population depletion. This view of fakery was too reflected in our study, with a comparatively greater proportion of the students categorised as Future Users, a crucial group in terms of forecasting China’s future consumptive trends, referring to the risk of fakes as an applicable barrier to this intended future consumption, relative to the other User Groups. What’s more, 66% of respondents who, at the time of the survey, were not engaged in wildlife product use (in other words, Former, Future and Non-Users) cited already using non-wildlife products with a similar function to wild-sourced products as a scenario applicable to this non-consumption, and Future Users to a comparatively greater extent. Future Users were also the user group with the highest proclivity to try substitutes for the first time. Bearing witness to this, substitutes being a safer and healthier option were some of the primary reasons respondents in our survey cited for choosing to adopt substitutes over wildlife. Chen (2017) economic model revealed that, for conservation purposes, it may be more beneficial to incentivise biotech firms producing synthetic rhino horn to produce inferior fake synthetic rhino horns (as oppose to marketing them as ‘perfect substitutes’ or ‘superior substitutes’ to wild horns) that are engineered to be undesirable in some respect but difficult for buyers to distinguish from wild horns through adverse selection/asymmetrical information. This model works on the postulation that the presence of inferior yet indistinguishable substitutes in the market creates buyer uncertainty of the quality of goods on sale and as a result, reduces buyers’ willingness-to-pay for horns, which then tends to put downward pressure on rhino horn price and hence lower the supply of wild horn.

The majority of our survey participants thought that providing wildlife substitutes priced considerably cheaper than wild-sourced would be useful or have some impact in mitigating wildlife product use. This notion was reiterated, with ‘substitutes are cheaper’ the greatest incentive for choosing alternatives amongst respondents. In addition, the survey revealed higher-priced goods weren’t frequently thought of as greater quality or potency. This could be indicative of the consumers not being so willing to pay a higher price for a wildlife product, instead price-conscious consumers. As such, they are more likely to purchase a cheaper priced substitute product provided it is perceived as good quality, since they are less likely to view such a substitute as sub-standard, as evidenced in this survey. What’s more, ‘conservation consciousness’ (balancing the ecosystem/ preventing extinction) emerged as another significant factor contributing to consumers choice to adopt substitutes in our study, mirroring Liu *et al.* (2016) findings. Indeed, Venkataraman's survey (2007) demonstrated Vietnamese university students to be the age group most willing to consider substitutes and alternatives to wildlife products.

Although there was a high overall rate of potential substitute acceptance, varying degrees of substitutability were revealed, dependent on the nature of the proposed substitute product. That is, synthetic wildlife substitutes, and to a lesser extent, farmed wild animals were both the most tried and preferred types of substitutes amongst respondents. This finding was in line with Liu *et al.* (2016) stated preference experiment whereby, out of the respondents who were willing to choose substitutes, synthetics were preferred over any other sources of available substitutes. Furthermore, our survey revealed fur to be the most substitutable wildlife product, indicated by synthetic fur being the type of substitute most widely accepted (90%). This is postulated to be, in part, due to respondents’ familiarity with this item, owing to its suitability to the region’s cold climate. Indeed, redirecting consumers’ behaviour towards a familiar or existing alternative rather than having to create a new behaviour, or having to stop this behaviour altogether, is less of a sizable jump, from a behavioural change standpoint (Burgess, 2016; Wallen & Daut, 2018).

Students’ education was the most influential source of information when it came to their familiarity with wildlife alternatives and the internet and social media another strong channel for conservation communication in this younger age bracket. Nonetheless, greater consumer exposure is needed to instil the view that substitutions are a choice that consumers have at their disposal, particularly vital to act upon at a time of widespread public sensibility to this topic in the wake of the COVID-19 pandemic. This enhanced awareness, enabling consumers to make an informed decision knowing all the facts, would be conducive to future uptake of substitutes.

## Conclusions and Recommendations for Further Research

The formative research we present here helps to update and advance our understanding of the prevalence of demand for wildlife products in our Chinese millennial student population, with particular consideration given to the dynamics of such demand. Specifically, how demand may evolve as this generation ages and their incomes increase (based on examining the underlying motivations that influence their consumptive choices). Of particular significance is our user sub-groups’ attitudes towards – and intentions to use – substitutes, which offers new and actionable insight into the potential uptake of substitutes, not as a panacea but nonetheless a viable intervention tool to explore further, moving forward in a post-pandemic world.

When interpreting our study’s findings, it is worth noting that over half of this survey’s respondents (53%) revealed a tendency to not consider the wild animal when consuming its associated product(s). This finding gives us reason to believe these millennials may not necessarily always associate their own personal consumption with the use of wildlife derivatives, resulting in possible under-reporting (inadvertent or deliberate) (Coals *et al.,* 2020; Zhang *et al.,* 2008). Indeed, Liu *et al.* (2016) found that when making purchasing decisions, respondents paid less attention to the composition of Traditional Asian Medicines (TAM), in comparison to TAM’s curative effects and function. This naturally casts doubt on the veracity of self-reported measures of consumption in determining to what extent these Chinese students are contributing to wildlife product demand. As highlighted by Davis *et al.* (2019), to obtain a more accurate reflection of actual consumption, future studies could utilise specialised questioning techniques to further counteract the issue of social desirability and preference falsification. Secondly, based on the ‘demand datapoints’ for evaluating end-market interventions proposed by Broad and Burgess (2020), our predominantly consumer opinion-based data could be complemented by triangulation against wider-market analytics. For instance, extending the survey to encompass qualitative inputs from key informants such as traditional medicine practitioners and retailers of bio-fabricated substitutes of wildlife products to capture retailer-opinion data: namely how retailers forecast future demand in this contemporary generation (based on the retailer’s understanding of their products’ customers). Where substitutes have been identified, understanding the volume of sales to Chinese millennials from retail outlets could also be beneficial to contextualise. Insights from ethnographic studies employing social listening and transaction observations in a TCM practice could also be a logical progression of our current work, in order to gather consumer observational data on situational prompts, narratives around customer queries and tag-words associated with consumer purchasing behaviour (Broad & Burgess, 2020).

Our study’s non-probabilistic approach means we cannot robustly extrapolate findings from our sample, beyond population-level generalisations, to be representative of the wider Chinese millennial cohort. Nonetheless, this study provides current information that lays the foundations for a fuller understanding of this emergent consumer group’s role in wildlife product consumption, upon which we can build further target audience insight, and potentially cover other second-tier Chinese cities. In this sense, our findings serve as a precursor to help inform the design and implementation of context-specific behavioural change interventions to halt a further surge of demand, and guide the future framing of product substitution marketed at this demographic. Although our study does not support the notion (due to our sample’s impetus to not use wildlife and to adopt substitutes, being rooted in personal principles more than temporary barriers), previous findings indicate wildlife product substitutes are a cheaper, entry-level product, whose widespread availability has not satiated consumer demand. Instead, in the case of farmed bear bile, Davis *et al.* (2019) postulates that the substitution effect acts to normalise prevalent consumption of species and may lead consumers to later seek out wildlife products. Hence, further research should consider in greater detail the longer-term outcomes that would likely follow a marked uptake of substitutes in this contemporary audience.

## Supplementary Information

Below is the link to the electronic supplementary material.

Supplementary file1 (DOCX 144 KB)

## Data Availability

The authors confirm that the data supporting the findings of this study will be made available on reasonable request. In addition, we intend to archive our anonymised data on the data repository the Open Science Framework at the time of publication.
