# Supplementary material for: Can Substitutes Reduce Future Demand for Wildlife Products: A Case Study of China’s Millennial Generation
Source: Hum Ecol Interdiscip J. 2021 Nov 6;50(1):91–111. doi: 10.1007/s10745-021-00279-0 (PMC8572063; doi:10.1007/s10745-021-00279-0)
Supplement: Supplementary file 1 — Supplementary file1 (DOCX 144 KB) [file 10745_2021_279_MOESM1_ESM.docx]

**Appendix A**

| **Table A:1**. Summary of survey respondent demographic characteristics (*n =* 350). | | | |
| --- | --- | --- | --- |
| **Variable** | **Category** | **Frequency** | **Percentage** |
| **Sex** | Male | 183 | 53.0 |
|  | Female | 162 | 47.0 |
|  | Prefer not to say | 5 | ___ |
| **Year of university study** | 1^st^ & 2^nd^ year | 108 | 31.6 |
|  | 3^rd^ & 4th year | 145 | 42.5 |
|  | Postgraduate student | 88 | 25.9 |
| **Type of Hometown** | Urban | 259 | 79.9 |
|  | Rural | 65 | 20.1 |
| **Travelled outside of China** | YES | 84 | 25.1 |
|  | NO | 251 | 74.9 |
| **Ethnicity** | Han | 290 | 88.7 |
|  | Manchu | 14 | 4.3 |
|  | Hui | 4 | 1.2 |
|  | Mongol | 7 | 2.1 |
|  | 9 other ethnic groups: Bouyei (3), Zhuang (2), Tibetan (2), Uyghur (2), Chuanqing (1), She (1), Yi (1), Miao (1), Geloa (1). | 14 | 4.3 |
| **Region of China respondent originates from** | **Northeast:**  Liaoning, Jilin, Heilongjiang, Tianjin | 87 | 28.6 |
|  | **North:**  Hebei, Shanxi, Beijing, Ningxia, Inner Mongolia, Gansu | 89 | 29.3 |
|  | **East:**  Shandong, Anhui, Jiangsu, Zhejiang | 52 | 17.1 |
|  | **West:**  Xinjiang, Qinghai, Guizhou, Tibet | 23 | 7.6 |
|  | **Central – south:**  Henan, Sichuan, Hunan, Yunnan, Jiangxi, Fujian, Hainan, Guangdong, Guangxi, Hubei | 53 | 17.4 |
| **Location of study** | Harbin | 244 | 69.5 |
|  | Beijing | 106 | 30.5 |
| **Age** | 18 - 21 | 160 | 46.9 |
|  | 22 - 25 | 157 | 46.0 |
|  | 26 - 31 | 23 | 6.9 |
| **Monthly income** | < 2000 yuan | 262 | 81.4 |
|  | > 2000 - 6000 yuan | 43 | 13.4 |
|  | > 6000 – 10,000 + yuan | 17 | 5.3 |
| **Programme of study** | Life & Physical Sciences | 105 | 31.7 |
|  | Engineering & Technology | 116 | 35.0 |
|  | Social Science & Humanities | 51 | 15.4 |
|  | Medicine (Human) | 59 | 17.8 |

| **TABLE A:2.**  **Respondent ID** | **User Group** | **Respondents’ open question responses when asked if there’s any other attributes of wildlife products that they value.** |
| --- | --- | --- |
| NEFU1 | Past & Future | “We can use [wildlife] because they have value and are very useful” |
| NEFU81 | Past & Future | “Wildlife products have significant effect in the treatment of certain diseases, but there is nothing special about decoration”. |
| NEFU99 | Past & Future | “They [wildlife products] are not necessities in life, and I support farmed products but against wildlife products”. |
| NEFU103 | Past & Future | “It has high value” |
| NEFU119 | Past & Future | “It is beneficial for health so we can use reasonably” |
| NEFU143 | Past & Future | “Everyone should value them because they are very precious” |
| NEFU154 | Past & Future | “In terms of benefits to humanity, we should explore wildlife [as a resource] reasonably but it is false if it is only for meeting people’s desires”. |
| NEFU171 | Past & Future | “Although wildlife products appeal to me to some degree, I can't accept them at the expense of nature” |
| BUCT264 | Past & Future | “Some products exactly attract me, but just as much as food and leather products” |
| BUCT271 | Past & Future | “I think wildlife products have high value” |
| NEFU340 | Past & Future | “It is forbidden to use animals from wild sources” |
| NEFU13 | Lapsed | “Wildlife products have advantages and disadvantages got humans” |
| NEFU17 | Lapsed | “It is correct only if the utilization is legal” |
| NEFU19 | Lapsed | “They are very important for medicine and have some aesthetic value” |
| NEFU24 | Lapsed | “Wild animals are still used as little as possible because they are a natural treasure” |
| NEFU46 | Lapsed | “I support medicinal wildlife products but don’t care about decorative wild animal products” |
| NEFU85 | Lapsed | “I do not favour the wildlife products very much but wildlife products are still sought after by some people, so they may not choose the artificial substitute” |
| NEFU89 | Lapsed | “Be against with using wildlife products” |
| NEFU123 | Lapsed | “I am very reluctant to use wildlife products, and they can't bring a sense of superiority” |
| NEFU147 | Lapsed | “Wild animals may have more or less value in medicine and aesthetics. But for the public, whether it is humane is the most worthy of attention”. |
| NEFU150 | Lapsed | “although I am not very familiar with wildlife products, I don't agree with wildlife trade using a variety of means” [as in even if legal] |
| NEFU174 | Lapsed | “I don't agree with fur coats, pangolins etc.” |
| HUCM221 | Lapsed | “We can use reasonably” |
| HUCM225 | Lapsed | “The wildlife products shouldn't be sold in the market and they should be protected illegally” [by being made illegal] |
| BUCT253 | Lapsed | “I do not agree with using or purchasing wildlife products” |
| BUCT336 | Lapsed | “I think wildlife animals are the same as farmed animals” |
| NEFU4 | Future | “They [wildlife] are very precious but they can be used reasonably” |
| NEFU8 | Future | “We should use reasonably/rationally and conserve effectively” |
| NEFU20 | Future | “They are legal and so can be used” |
| NEFU21 | Future | “They are very advanced and stylish” |
| NEFU26 | Future | “It can form an industry, but there is no need to kill” |
| NEFU35 | Future | “Resist as [and when] we can” |
| NEFU97 | Future | “Wildlife products have a certain effect, but should not be overly sought after” |
| NEFU192 | Future | “I really hate wildlife products” |
| BUCT268 | Future | “It may cause the wildlife to go extinct” |
| BUCM276 | Future | “We shouldn't use [wildlife] except for urgent things, such as medicine and cultural inheritance” |
| BUCM309 | Future | “university students do not have the ability to pay for the wildlife products, so you should widen the scale of this survey |
| NEFU15 | Non-User | “Although I think the wildlife products are very nice, I will not buy them!” |
| NEFU55 | Non-User | “It is very inhumane to purchase and obtain wildlife products” |
| NEFU129 | Non-User | “People live in harmony with animals” |
| NEFU137 | Non-User | “We should conserve them to breed and grow when producing wildlife products” |
| NEFU145 | Non-User | “I am not familiar with wildlife products and I don't think they are a necessity in our lives. We should conserve them, not use them. I think the most attractive thing about wildlife is their rarity and practicality. In today’s technologically advanced world, it's practicality can be artificially created and replaced, and its rarity is just a way for the rich to pursue luxury. We can conduct education to improve people's awareness of wildlife conservation”. |
| NEFU158 | Non-User | “I think the farmed animals can meet people’s needs all in all - I think most of the consumers are the middle-aged” |
| NEFU162 | Non-User | “Wildlife are exactly very precious, unlike domestic animals. Although they indeed have benefits and are very unique, we also should not hunt them just for business and we should balance the ecosystem” |
| HUCM214 | Non-User | “I don't agree with using wildlife products” |
| HUCM230 | Non-User | “In ancient times, wild animals were often associated with the words ‘right’ and ‘power’. Today, with the development of science and technology wildlife has healthier characteristics, especially in medicine aspects. But we also have to work hard to develop substitutes to better conserve wildlife”. |
| BUCT233 | Non-User | “No business, no harm!” |
| BUCT236 | Non-User | “Using wildlife will break the balance of nature” |
| BUCT239 | Non-User | “For the wildlife consumption, I think it is not only the atmosphere of society but also one's mentality that all drives consumption. So, I suggest that you can study the consumers psychology”. |
| BUCT243 | Non-User | “Wildlife also have life as do humans, and they have their rights to live with people” |
| BUCM289 | Non-User | “Not very interested in [wildlife products]” |
| BUCM297 | Non-User | “you can widen the scale of this study because the thoughts of students may be more traditional” |
| BUCM302 | Non-User | [In order] “to conserve the diversity of wildlife I object to using wildlife products” |
| BUCT324 | Non-User | “Very expensive and useless” |
| BUCT333 | Non-User | “there is no special meaning about wildlife products” |
| BUCT337 | Non-User | “I am not familiar with wildlife products” |
| NEFU342 | Non-User | “I am not interested in wildlife products unless some urgent things, such as saving lives” |

**Appendix B**

[
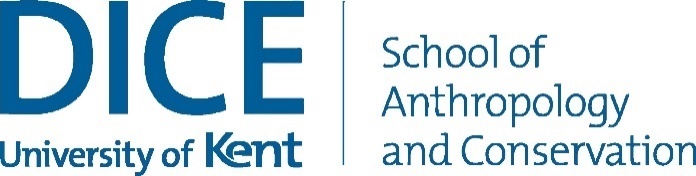
](https://www.google.com/url?sa=i%26rct=j%26q=%26esrc=s%26source=images%26cd=%26cad=rja%26uact=8%26ved=2ahUKEwi57fqo5priAhXwyIUKHZ_RBjUQjRx6BAgBEAU%26url=http%3A%2F%2Fwww.birdsontheedge.org%2F2017%2F04%2F14%2Fwant-to-study-choughs%2F%26psig=AOvVaw0EnREcyJDu_grIz8iP3zeY%26ust=1557915679069635)**Participants Information Sheet**

**Title:** The utilisation of and perspectives on wildlife products amongst the younger generation in China.

Conducted as part of my Master’s research project, sponsored by the University of Kent.

**Objectives of research:**

This study is investigating Chinese millennials’ attitudes and preferences regarding using wildlife products and their associated substitutes. To participate, you should be a student at a university in Harbin or Beijing, and born between 1989-2001.

None of participants information will be disclosed or used without their consent under Chinese Statistics Law. The results (excluding personal information) may be published in academic journals. Participants are welcome to include their email at the end of the questionnaire if they would like to be contacted with the results of the study.

This research project has been funded by the University of Kent, Canterbury, United Kingdom and has been approved by the University of Kent’s Ethics Committee in accordance with the Code of Ethical Practice for Research. This study was organized with the support of Northeast Forestry University, Harbin, China.

If you have any questions or would like more information on the purpose of this study, please email the researcher at kiir2@kent.ac.uk.

Thank you for your support and cooperation in this study.

Yours Sincerely,

Miss Katherine Rock,

MSc student

University of Kent

Email: [kiir2@kent.ac.uk](mailto:kiir2@kent.ac.uk) Phone: +44 7919948417

**Title of project**: Investigating the potential efficacy of substitutes in reducing demand for wildlife products in Chinese trade: A case study of the millennial generation.

**Name of investigator:** Miss Katherine Rock

**Participant Identification Number for this project:**

| 1. I confirm I have read and understood the participant information sheet for the above study. I have had the opportunity to consider the information, ask questions and have had these answered satisfactorily. |  |
| --- | --- |
| 1. I understand that my participation is voluntary and that I am free to withdraw at any time without giving any reason. Please email [kiir2@kent.ac.uk](mailto:kiir2@kent.ac.uk) with your participation identification number before 10^th^ July 2019 to be removed from the study. |  |
| 1. I understand that my responses will be anonymised before analysis. I give permission for members of the research team to have access to my anonymised responses. |  |
| 1. I agree to take part in the above research project. |  |

**Questionnaire Instructions:**

There are no right or wrong answers in this questionnaire. You only need to select the option that best describes your situation or opinion or give your own answer in the space provided.

To help you understand the questions, we’ve provided below some definitions of terminology included in the questionnaire:

**(1) Wildlife products:** – any product derived from non-domesticated animals or plants extracted from their natural environment. It can involve the trade of living or dead individuals, tissues such as skins, bones or meat, and other products.

**(2) Types of wildlife products** **addressed in this questionnaire:** -

1. Using wild animals as **food**
2. Using **medicine or tonic products** containing wildlife as an ingredient
3. **Ornamental items and garments** made from wildlife e.g. fur pelts

**(3) Wildlife substitutes:** Their purpose is to re-direct demand away from the original supply of conservation concern (due to overharvesting) towards a comparable product with equivalent quality; providing respite to facilitate wild population recuperation.

**(4) Types of substitutes:**

1. Same species from other countries (where abundant, sustainably harvested)
2. Captive production/ wildlife farming or artificial propagation
3. A different wildlife species altogether (with similar perceived quality/utility)
4. Domesticated animal or plant species
5. Inorganic or synthetic/bio-fabricated replacement

**PART A: CONSUMPTION OF WILDLIFE GOODS**

**1) Have you ever participated in any of the following activities?**

(Tick *ALL* that apply)

- Visited a wildlife market
- Eaten wildlife
- Hunted wildlife
- Seen wildlife in the zoo, entertainment e.g. circus
- Other similar activity (*please specify*) ______________________________________

**2) Have you ever used or purchased a wildlife product in the past or aspire to use in the future?**

(Tick *ONE* statement below that most accurately describes your situation)

1. ◻ I use wildlife products on a regular basis, including in the last six months.
2. ◻ I have used wildlife products before and intend to use them in the future.
3. ◻ I have used wildlife products in the past but do not intend to use them in the future
4. ◻ I am yet to use any wildlife products but would consider trying them out in the future.
5. ◻ I have never used any wildlife products and have no intention to in the future.

If you answered **v to Q 2**, please skip to **Q 5 (PART B)**

**3) Out of the wildlife product categories below, please fill in *Table 1* according to the following instructions: -**

Table 1 below contains categories of wildlife products. Please rank how likely you are to use or purchase each product category in column 2 (on a scale of 1 – 3, 1 being most likely, 3 being least likely) and give an example of your preferred product choice in column 3.

| **Wildlife product category** | **Rank in order of how likely you are to use this product category (1 -3)** | **Example of preferred wildlife product** |
| --- | --- | --- |
| **Food** containing wildlife parts  e.g. pangolin meat |  |  |
| **Medicine** with wildlife as an ingredient(s) e.g. musk in Huo xue zhi tong cha ji for migraines. |  |  |
| **Ornaments/fashion** containing wildlife materials  e.g. fur coat, necklace |  |  |

**PART B: ATTITUDE TOWARDS WILDLIFE PRODUCTS**

**4) For each statement below, please indicate to what extent you agree or disagree.**

|  | Strongly agree | Agree | Neither agree nor disagree | Disagree | Strongly disagree |
| --- | --- | --- | --- | --- | --- |
| I find wildlife products that are exclusive or highly sought-after desirable, even if they are harder to get hold of. For instance, something from an extinct or rare, precious species. |  |  |  |  |  |
| Wildlife products sourced from their natural environment are more desirable as they are of authentic origin, more potent or of greater purity. |  |  |  |  |  |
| I would order wild meat over farmed meat in a restaurant, even if it’s more expensive as its more delicious or creates more of an impression amongst guests. |  |  |  |  |  |
| Imitations of wildlife products are an inferior choice to wild-caught. |  |  |  |  |  |
| A higher price reflects the products greater value and superior quality or curative potency. |  |  |  |  |  |
| Wildlife products of spiritual significance resonate most with me. I am drawn to items that bring good fortune and protection to business or personal life |  |  |  |  |  |
| Using wildlife products symbolises wealth, sophistication or success and enhances my image and reputation. Strengthens business exchanges and respect from others. |  |  |  |  |  |
| I believe consuming products derived from a powerful, strong animal will lead me to imbue such attributes, embody their virtues. |  |  |  |  |  |
| I value wildlife products for their aesthetic or decorative qualities. They can create a good Feng Shui and enhance the interior design. |  |  |  |  |  |
| I value wildlife products for their health benefits, curative effect to treat illness and promote wellness**.** Wild sourced products have greater medicinal strength. |  |  |  |  |  |
| Wild caught animal products are more nutritious and healthier than farmed. |  |  |  |  |  |
| Wildlife products are a financial asset worth collecting for their market value. |  |  |  |  |  |
| I would purchase wildlife products as a gift to a valued business associate or close personal friend to show my respect and gratitude. |  |  |  |  |  |
| A wildlife product that epitomises tradition and celebrates cultural heritage appeals to me. |  |  |  |  |  |
| Purchasing prestigious wildlife goods is a way to express individuality |  |  |  |  |  |
| On the whole, I find wild animal goods intriguing and desirable. |  |  |  |  |  |

Any other things you value in wildlife products? (*Please specify below*)

_______________________________________________________________

**5)– If you are not currency using wildlife products, please indicate your reasons.**

For each reason below, indicate if it applies to why you choose not to use or purchase wildlife species:

| *Reasons stopping you from using wildlife products …* | Definitely applies | Usually applies | Don’t know | Rarely applies | Definitely doesn’t apply |
| --- | --- | --- | --- | --- | --- |
| Discouraged by high prices |  |  |  |  |  |
| Animal welfare concerns |  |  |  |  |  |
| Not worth using as benefits are questionable |  |  |  |  |  |
| I use non-wildlife products with a similar function instead |  |  |  |  |  |
| Risk of penalties |  |  |  |  |  |
| Risk of buying a fake too high |  |  |  |  |  |
| Already have enough wildlife products |  |  |  |  |  |
| Ashamed to buy |  |  |  |  |  |
| My relatives/friends disapprove |  |  |  |  |  |
| Saving it for a future special occasion e.g. wedding. |  |  |  |  |  |
| Have attempted to use but scarce availability |  |  |  |  |  |
| Not interested or never had the opportunity |  |  |  |  |  |
| Don’t believe wildlife products should be used |  |  |  |  |  |
| Hygiene or health risks (zoonotic disease) |  |  |  |  |  |

Other reason (*please specify*) _______________________________________________________________

**6) Are there any wildlife species you think should not be used to produce wildlife products?**

Wildlife species name ___________________________________________________________________________

If so, why is this the case? (Tick *ALL* that apply)

- I do not purchase or use species that are a national symbol of China
- I do not purchase or use species that I think are cute or attractive.
- I do not purchase or use species that are protected under National Grade 1 and/or 2 status.
- I do not purchase or use species that I think of as ‘dirty’ or unhygienic.
- I tend not to consider the wild animal when buying or using the product.
- I only purchase farmed wild animals.
- Any other reason (*please specify*) __________________________________________________________________________________________________________________________________________________________________________________________________________

**7) Please specify what kind of wildlife you think should be or shouldn’t be used by humans?**

Could be used: Species 1 ________________________ Species 2 ______________________

Shouldn’t be used: Species 1 _______________________ Species 2 _______________________

**8) When should wildlife consumption be allowed? Please indicate to what extent you agree or disagree with each statement below.**

| *Should wildlife consumption be allowed under any circumstances?* … | Strongly agree | Agree | Neither agree nor disagree | Disagree | Strongly disagree |
| --- | --- | --- | --- | --- | --- |
| Yes, humans have a right to use animals as a natural resource for our own benefit |  |  |  |  |  |
| Yes, but only if the given wild animal product has a legitimate and proven benefit. |  |  |  |  |  |
| Yes, but only sparingly for special occasions like New Year, weddings. |  |  |  |  |  |
| Yes, but only in the case of an emergency e.g. poor health, last resort. |  |  |  |  |  |
| Yes, but only for wild animals that are plentiful. |  |  |  |  |  |
| Not if the animal is threatened by extinction or protected. |  |  |  |  |  |
| No, wildlife should never be used for human benefit, species have an intrinsic right to co-exist with humans. |  |  |  |  |  |
| I don’t know enough about this subject to confidently answer this question |  |  |  |  |  |
| I am indifferent either way. |  |  |  |  |  |

Any other comment you wish to make? (*please specify*) ________________________________________________________­­­­___________________

**9) Indicate below how useful you think each strategy would be in mitigating unsustainable harvesting of wildlife.**

| Strategy | Useful | Some impact | Not useful |
| --- | --- | --- | --- |
| Greater regulation and enforcement of legislation |  |  |  |
| Social pressure that leads to a shift away from the desire to use wildlife products |  |  |  |
| Provision of wildlife substitutes/alternatives that are considerably cheaper |  |  |  |
| Protecting more wildlife species, even if this means restriction of the use of these species. |  |  |  |
| Educating people about the risk of zoonotic disease and other health problems associated with wildlife consumption. |  |  |  |

Other (*please specify*)

___________________________________________________________________________

**PART C: SUBSTITUTES FOR WILDLIFE PRODUCTS**

**10) Tick *ONE* statement below that best describes your opinion on substitutes: -**

◻ I have used wildlife product substitutes several times before or in the last month

◻ I had a positive experience with wildlife substitutes before so will continue to use them.

◻ I had an unsatisfactory experience with wildlife substitutes in the past, so I don’t intend to use them in the future. Instead I will continue to use wild sourced products.

◻ I have not yet used any wildlife substitutes but am willing to try.

◻ I have not used wildlife substitutes and do not intend to in the future. I believe wildlife products are irreplaceable.

**11) If you have tried a wildlife product substitute before, please specify:**

1. Name of *substitute* you’ve used most recently: _______________________________________________
2. Type of substitute:

- Farmed/captive
- A different wildlife species from my usual
- Synthetic material
- Domesticated animal

1. Reason for using wildlife product substitute instead of original wildlife product: _______________________________________________________________________________________________________________________________________________________________________________________________________________________________________________________________________________________

**12) How did you hear about wildlife substitutes?**

Tick boxes for all options in Table 6 below that apply to you. Of those selected, rank each option according to its importance in influencing/ introducing you to wildlife substitutes (1 being the most influential to your decision making, 8 being the least influential). For the options that don’t influence you, please leave blank.

| **Reasons for your familiarity with wildlife substitutes** | **Importance in influencing you (1 - 8)** |
| --- | --- |
| ◻ My education |  |
| ◻ Recommended by family member |  |
| ◻ Saw on internet e.g. social media |  |
| ◻ Recommended by a TCM practitioner or pharmacist |  |
| ◻ Recommended by a friend or co-worker |  |
| ◻ Television or radio advert |  |
| ◻ No one influenced me, I sought it out on my own |  |
| ◻ I am more of an influence on others regarding alternatives to wild animal products than they are on me |  |

Other reason (*please specify*) ______________________________________________________________________________

**13) Tick boxes for all wildlife products in Table 7 below that you would be willing to try at least one of the available substitutes. Of those selected, rank each wildlife product according to how likely you are to try out the substitutes (on the scale of 1 - 6, 1 being most likely, 6 least likely). Then select *ONE* wildlife product from the list below (mark with an X) - The researcher will provide the corresponding information sheet for your choice.**

| **Wildlife product. Tick if you are open to try out its substitutes** | **Available substitutes** | **How likely are you to try out substitutes?**  **(rank 1-4)** | **Chosen option (X)** |
| --- | --- | --- | --- |
| - Natural Fur | - Faux fur , faux leather |  |  |
| - Deer musk | Farmed musk deer, Synthetic musk, Plant substitute : *Acorus tatarinowii* , Animal substitute : Muskrat, musk ox |  |  |
| - Sea food | Abalone : plant substitute (konjaku, konnyaku *Amorphophallus konjac*) |  |  |
| - Velvet deer Antler | Farmed, Ginseng substitute, Synthetic |  |  |

Other wildlife products for which you would be prepared to try out substitutes (*Please specify*) ____________________________________________________________________________________________________________________________________________________________________

**PART E: SOCIO-DEMOGRAPHIC INFORMATION**

**(1)** Gender: ◻ Male ◻ Female ◻ Prefer not to say

**(2)** Age: ____________________

**(3)** Highest level of education: ◻ Junior High School ◻ Senior High School ◻ College diploma ◻ Undergraduate (*if so, please circle which year*): Year 1, Year 2, Year 3, Year 4, ◻ Postgraduate (*if so, please indicate what kind*): Masters, PhD ◻ Other (*Please specify*) __________________

**(4)** Programme of study (*if currently a student*) ___________________________

**(5)** Location of family residence (*province/municipality*) ___________________

Would you describe your hometown as: ◻ City/ urban area ◻ Town or suburb ◻ Rural area

**(6)** Parents profession: ___________________________­­­­­­­­­­­­­­­­­­_______________________________________________

**(7)** Ethnicity: ____________________________________________

**(8)** Which of these bests describes your occupation (*please tick ONE*)

◻ Full-time employment ◻ Part-time employment ◻ Full-time education ◻ Part-time education ◻ Unemployed and seeking work ◻ Unemployed not seeking work ◻ Other (*please specify*)

**(9)** Which of these bests describes your *monthly* income (if applicable):

◻ < 2000 yuan ◻ 2000 - 4000 yuan ◻ 4000-6000 yuan ◻ 6000 – 8000 yuan ◻ 8000 – 10,000 yuan ◻ > 10,000 yuan

**(10)** Have you ever travelled outside of mainland China (*please tick one*) ◻ YES ◻ NO

**(11) Having completed this questionnaire, do you have any further comments?**

______________________________________________________________________________________________________________________________________________________________________________________________________________________________________________________

**(12) Thank you for your time and participation in the study, it is greatly appreciated.** Please add your email below if you’d like to be contacted with the findings of this research: **__________________________________________________________________________________**

**FUR**

**1) Have you worn or owned any fur products?**  ◻ YES ◻ NO

**2) Would you consider this item…**

◻ a luxury good ◻ an essential item **or** ◻ Unnecessary *(Please tick ONE*).

**3) What occasion would warrant wearing fur?**

- Only special occasions e.g. weddings, gifted
- Business, professional events
- Colder climate in winter
- No special occasion is required

**4) To what extent do you agree or disagree with the following statements:**

*(1) ‘The skins of wild animals should not be made into fur coats.*

◻ Strongly Agree ◻ Agree ◻ Neither agree nor disagree ◻ Disagree ◻ Strongly disagree

*(2) ‘Breeding animals for their skins is a legitimate use of animals’* (Davey, 2006).

◻ Strongly Agree ◻ Agree ◻ Neither agree nor disagree ◻ Disagree ◻ Strongly disagree

**5) What attributes of natural fur appeal most to you?**

| **Attribute** | Very important | Important | No opinion | Not so important | Not important at all |
| --- | --- | --- | --- | --- | --- |
| Uniqueness, rarity |  |  |  |  |  |
| Wild-caught origin as more authentic |  |  |  |  |  |
| High price reflecting exclusivity and luxurious quality |  |  |  |  |  |
| Aesthetic image enhancer, fashion statement, symbolic |  |  |  |  |  |
| A collectible and investible item |  |  |  |  |  |
| Suitability as a gift/gesture |  |  |  |  |  |
| Epitomises Chinese tradition |  |  |  |  |  |
| Longevity and durability |  |  |  |  |  |

**6) Would you ever consider switching to a fur replacement/ alternative?**

(1) Farmed fur ◻ YES ◻ NO

(2) Synthetic ‘faux’ fur ◻ YES ◻ NO

(3) No, wild fur is irreplaceable; I will only accept genuinely real fur ◻

**DEER MUSK**

**1) Have you worn or owned any deer musk products?**  ◻ YES ◻ NO

**2) Would you consider this item…**

◻ a luxury good ◻ an essential item ◻ Unnecessary *(Please tick ONE*).

**3) What occasion would warrant using deer musk?**

- Only special occasions e.g. weddings, gifted
- Business, professional events
- No special occasion is required

**4) To what extent do you agree or disagree with the following statements:**

*(1) ‘Musk is only authentic if farmed from a wildlife species but doesn’t have to be musk deer’*

◻ Strongly Agree ◻ Agree ◻ Neither agree nor disagree ◻ Disagree ◻ Strongly disagree

*(2) ‘Musk deer should be farmed sustainably even though this results in not satisfying the demand for this product’*

◻ Strongly Agree ◻ Agree ◻ Neither agree nor disagree ◻ Disagree ◻ Strongly disagree

*(3) ‘Ambergris* (*Lóng xián xiang*) *is an alternative musk sourced from sperm whales. I would try this as its harvest does not harm the wildlife species’*

◻ Strongly Agree ◻ Agree ◻ Neither agree nor disagree ◻ Disagree ◻ Strongly disagree

**5) What attributes of deer musk appeal most to you?**

| **Attribute** | Very important | Important | No opinion | Not so important | Not important at all |
| --- | --- | --- | --- | --- | --- |
| Uniqueness, rarity |  |  |  |  |  |
| Wild-caught origin as more authentic |  |  |  |  |  |
| High price reflecting exclusivity and luxurious quality |  |  |  |  |  |
| Aesthetic image enhancer, fashion statement, symbolic |  |  |  |  |  |
| A collectible/ investible item |  |  |  |  |  |
| Suitability as a gift/gesture |  |  |  |  |  |
| Epitomises Chinese tradition |  |  |  |  |  |

**6) Would you ever consider switching to a musk replacement/ alternative?**

(1) Farmed deer musk: ◻ YES ◻ NO

(2) Synthetic musk: ◻ YES ◻NO

(3) Wild species substitute: muskrat, civet, musk ox: ◻ YES ◻ NO

(4) Plant substitute (*Acorus tatarinowii):* ◻ YES ◻ NO

(5) Ambergris ("Lóng xián xiang") from sperm whale: ◻ YES ◻ NO

**ABALONE**

**1) Have you consumer or owned any abalone before?**  ◻ YES ◻ NO

**2) Would you consider this item…**

◻ a luxury good ◻ an essential item ◻ Unnecessary *(Please tick ONE*).

**3) What occasion would warrant using deer musk?**

- Only special occasions
- Business, professional events
- No special occasion is required

**4) What attributes of abalone appeal most to you?**

| **Attribute** | Very important | Important | No opinion | Not so important | Not important at all |
| --- | --- | --- | --- | --- | --- |
| Uniqueness, rarity |  |  |  |  |  |
| Wild-caught origin as more authentic |  |  |  |  |  |
| High price reflecting exclusivity and luxurious quality |  |  |  |  |  |
| Aesthetic image enhancer, symbolic |  |  |  |  |  |
| Suitability as a gift/gesture |  |  |  |  |  |
| Epitomises Chinese tradition |  |  |  |  |  |
| Taste |  |  |  |  |  |

**5) Would you ever consider switching to an abalone replacement/ alternative?**

(1) Farmed: ◻ YES ◻ NO

(2) Alternative seafood product (less rare): ◻ YES ◻ NO

(3) Plant substitute *Amorphophallus konjac* (Konjac): ◻ YES ◻ NO

**VELVET DEER ANTLER**

**1) Have you used or owned any velvet deer antler products?**  ◻ YES ◻ NO

**2) Would you consider this item…**

◻ a luxury good ◻ an essential item ◻ Unnecessary *(Please TICK ONE*).

**3) What occasion would warrant using deer antler?**

- Only special/rare occasions
- Business, professional events
- No special occasion is required

**4) What attributes of velvet deer antler appeal most to you?**

| **Attribute** | Very important | Important | No opinion | Not so important | Not important at all |
| --- | --- | --- | --- | --- | --- |
| Uniqueness, rarity |  |  |  |  |  |
| Wild-caught origin as more authentic |  |  |  |  |  |
| High price reflecting exclusivity/ luxurious quality |  |  |  |  |  |
| Aesthetic image enhancer, fashion statement, symbolic |  |  |  |  |  |
| A collectible/ investible item |  |  |  |  |  |
| Suitability as a gift/gesture |  |  |  |  |  |
| Epitomises Chinese tradition |  |  |  |  |  |
| Medicinal powers |  |  |  |  |  |

**5) Would you ever consider switching to a velvet deer antler replacement/ alternative?**

(1) Farmed: ◻ YES ◻ NO

(2) Ginseng substitute: ◻ YES ◻ NO

(3) Synthetic substitute: ◻ YES ◻ NO

[
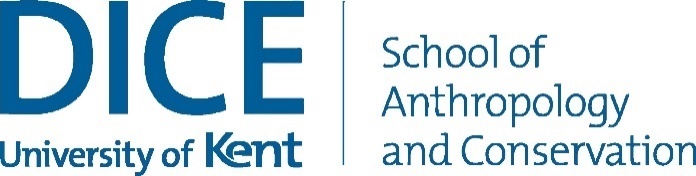
](https://www.google.com/url?sa=i%26rct=j%26q=%26esrc=s%26source=images%26cd=%26cad=rja%26uact=8%26ved=2ahUKEwi57fqo5priAhXwyIUKHZ_RBjUQjRx6BAgBEAU%26url=http%3A%2F%2Fwww.birdsontheedge.org%2F2017%2F04%2F14%2Fwant-to-study-choughs%2F%26psig=AOvVaw0EnREcyJDu_grIz8iP3zeY%26ust=1557915679069635)**参与者信息表**

**题目：哈尔滨市年轻一代对野生动物产品的使用和观点**

**研究目的：**

**本实验旨在研究哈尔滨市年轻一代对使用野生动物产品及其替代品的态度和偏好。参与者必须是哈尔滨市在1989-2001年出生的大学生。**

**研究说明：**

**本研究符合中国法律，参与者的任何信息都不会被泄露或使用。结果（不包括个人信息）会在学术期刊上发表。如果您希望了解本研究的结果，欢迎您在问卷末尾填写您的电子邮件，我们将会与您联系。**

**本次研究项由英国肯特大学资助，并依照“研究伦理实践准则”，已获得肯特大学伦理委员会的批准，本研究是在东北林业大学的支持下组织的。**

**如果您对本研究的目的有任何问题或想了解更多信息，请发送电子邮件至kiir2@kent.ac.uk，与本次研究人员联系。非常感谢您对本次研究的支持！**

**调查人员：**

**Katherine Rock, 英国肯特大学**

**Email: kiir2@kent.ac.uk**

**Phone: +44 7919948417**

**课题名称：**

调查替代品在减少中国贸易中对野生动物产品需求方面的潜在功效：千禧一代的案例研究。

**调查人员姓名：**Katherine Rock

本次参与者参与识别码：

| 1. 我确认已阅读并理解上述的参与者信息表，并且我有机会考虑这些信息，提出问题并得到了令人满意的答复。 |  |
| --- | --- |
| 1. 我理解我的参与是自愿的，我可以随时自由抽回我的信息而不给出任何理由。若您要抽回您的信息，请在2019年7月10日之前将您的参与识别号码发送至kiir2@kent.ac.uk，以便从研究中删除。 |  |
| 1. 我知道在分析之前我的回复将被匿名化，我允许研究团队成员研究我的匿名答案。 |  |
| 1. 我同意参加上述研究项目。 |  |

**问卷中的问题没有对错之分，您只需要选出您认为最适合的答案**

**试验方法：**

问卷中的答案没有对错之分，您只需选择出最能描述您的情况或意见的选项，或在所提供的空白处提供您自己的答案。为帮助您更好的理解问卷，下面提供了一些问卷中提到的术语定义：

1. **野生动物产品**：来源于非家养动物或从其直接从自然环境中得到的植物，包括活体贸易或死亡的个体、皮肤、骨头、肉类等组织以及其他产品。
2. **本调查问卷中涉及的野生动物产品类型**
3. 食用野生动物
4. 使用含有野生动物作为成分的药物或滋补品
5. 利用野生动植物制成的装饰物品和服装，例如毛皮
6. **野生动物替代品：**使用替代品的目的是将需求从原始的野生动物资源（由于过度采伐的危险）转移到具有同等质量的同类产品，提供机会以促进野生数量的恢复。
7. **替代品的类型：**
8. 其他国家的同类物种（资源充足，可持续狩猎）
9. 资源生产、野生动物养殖或人工繁殖
10. 另一种完全不同的野生动物物种（具有相似的质量/效用）
11. 家养的野生动植物物种
12. 人工制造的替代品

**Part A：野生动物产品消费**

1. **您曾经参加过下列活动吗？（请选出所有您觉得符合的）**

🞎 参观野生动物市场

🞏 食用野生动物

🞏 野生动物狩猎

🞎 在动物园或其他娱乐场所参观野生动物，如马戏表演

🞎 其他的相似活动（请详细说明）

1. **您曾经购买或使用过野生动物产品吗？或是您未来想使用野生动物产品吗？（请在下面选择一个最符合您的情况的）**
2. 🞎 我定期使用野生动物制品，在过去六个月也曾使用过
3. 🞎 我曾经使用过野生动物制品，并且未来也会使用它们
4. 🞎 我曾经使用过野生动物制品，但未来并不打算使用
5. 🞎 我至今未使用过任何野生动物制品，但将来会考虑尝试着使用它们
6. 🞎 我未使用过任何野生动物制品，并且将来也不打算使用

【如果您问题2选择了⑤，请您直接从第5个问题开始回答（Part B部分）】

1. **在下面野生动物产品类别中，请按照以下说明填写表1**

表1中包含了三类野生动植物产品类别，请您根据使用或购买的可能性，对这三类野生动物产品进行排序（1代表最有可能，3代表最不可能）。并且请您在认为最有可能使用的野生动物产品后面（第3列）写出一个例子。

| **野生动物产品类别** | **按您使用或购买此类产品的可能性排序（1-3）** | **举例** |
| --- | --- | --- |
| 含有野生动物的食品，例如：穿山甲肉 |  |  |
| 用野生动物作为成分的药物，例如麝香可以活血止痛（治疗偏头痛） |  |  |
| 利用野生动物材料制作的装饰品或服装，例如毛皮大衣，项链 |  |  |

**Part B：对野生动物产品的态度**

1. **对于下表2中的每个陈述，请您在您认为合理的一项打上“√”**

|  | **非常同意** | **同意** | **无所谓** | **不同意** | **非常不同意** |
| --- | --- | --- | --- | --- | --- |
| 我认为野生动物产品是独一无二或备受追捧的，尽管它们很难得到。例如，来源于灭绝或珍贵稀有物种的制品。 |  |  |  |  |  |
| 源于自然环境的野生动物产品更为理想，因为它们具有真实性，更有效或纯度更高。 |  |  |  |  |  |
| 在餐厅吃饭时，尽管野生肉类更贵，但对于养殖肉类我还是倾向于点一些野生肉类，因为它更美味或能给客人留下更多印象。 |  |  |  |  |  |
| 野生动物产品的仿制品是野外捕获产品的劣质选择 |  |  |  |  |  |
| 野生动物产品价格越高，则代表它们具有更大的价值和更加卓越的品质或治疗效力。 |  |  |  |  |  |
| 具有精神意义的野生动物产品最能引起我的共鸣，我被那些可以给商业或个人生活带来好运和保护的产品所吸引。 |  |  |  |  |  |
| 使用野生动物产品象征着财富，成熟或成功，并且可以提升我的形象和声誉，也可以加强业务交流和赢得他人尊重。 |  |  |  |  |  |
| 我相信消费来自强大、有力量的动物的产品可以使我得到这些特点，体现了它们的美德 |  |  |  |  |  |
| 我重视野生动物产品因为它们的美学或装饰价值，他们可以创造一个好的风水和增强室内设计。 |  |  |  |  |  |
| 我重视野生动物产品因为它们对健康有益，可以促进健康，治疗某些疾病。并且野外资源的产品有更高的药用价值。 |  |  |  |  |  |
| 野外捕获的野生动物产品比养殖产品更加营养和健康 |  |  |  |  |  |
| 野生动物产品是一种具有市场价值，值得收藏的金融资产 |  |  |  |  |  |
| 我会购买野生动物产品当做礼物送给重要的商业合作伙伴或是亲密的朋友，以显示我对他们的尊重和感激 |  |  |  |  |  |
| 体现传统和文化遗产的野生动物产品更吸引我 |  |  |  |  |  |
| 购买有名的野生动物商品是表达个性的一种方式 |  |  |  |  |  |
| 总之，我认为野生动物商品很有趣，也很有吸引力 |  |  |  |  |  |

您对野生动物产品有何评价？ （请在下面说明）

1. **如果您没有使用野生动物产品（以前或是未来），请您对下表中的每个原因进行判断，标明其是否导致了您不使用或购买野生动物产品**

| **导致您不使用野生动物产品的原因** | **非常适用** | **一般适用** | **不知道** | **不太适用** | **绝对不适用** |
| --- | --- | --- | --- | --- | --- |
| 支付不起（因为高昂的价格退却） |  |  |  |  |  |
| 出于对动物福利的关心 |  |  |  |  |  |
| 不值得使用，因为好处是值得怀疑 |  |  |  |  |  |
| 我使用具有相同作用的非野生动物产品 |  |  |  |  |  |
| 有被处罚的风险 |  |  |  |  |  |
| 买到假货的风险太高 |  |  |  |  |  |
| 已经有足够的野生动物产品 |  |  |  |  |  |
| 对于购买感到惭愧 |  |  |  |  |  |
| 我的亲戚或朋友不同意 |  |  |  |  |  |
| 保存下来用在将来的一些特殊场合，比如结婚 |  |  |  |  |  |
| 曾经试图使用，但由于稀缺/有限的可得性 |  |  |  |  |  |
| 没有兴趣或没有机会 |  |  |  |  |  |
| 认为不应该使用野生动物产品 |  |  |  |  |  |
| 卫生或健康风险（人畜共患病） |  |  |  |  |  |

其他的原因，请具体写出

1. **您认为哪种野生动物不应该被用于当做野生动物产品？**

野生动物物种名称

是什么原因导致您产生了这种想法？（选出所有符合的）

🞎 我不使用或购买这类野生动物，因为它是中国的民族象征

🞎 我不使用或购买这类野生动物，因为我认为它很可爱、很吸引人

🞎 我不使用或购买国家一级/二级保护的野生动物

🞎 我不使用或购买这类野生动物，因为我认为它脏或不卫生

🞎 在购买或使用产品时，我不倾向于考虑野生动物

🞎 我只购买养殖的野生动物

🞎 其他原因（请具体写出）

1. **请列举出您认为哪种物种可以被人类使用，或不可以被人类使用？**

可以被人类使用：物种1 物种2

不可以被人类使用：物种1 物种2

1. **您认为什么情况下消费野生动物可以被允许？请在下表中标明您对每一条陈述的同意程度。**

| **在任何情况下都应该允许野生动物消费吗？** | **非常同意** | **同意** | **无所谓** | **不同意** | **非常不同意** |
| --- | --- | --- | --- | --- | --- |
| 是的，人类有权利为了自身的利益使用野生动物，他们是自然资源的一种 |  |  |  |  |  |
| 是的，但是只有当规定的野生动物产品具有合法且经证实的益处时才可使用 |  |  |  |  |  |
| 是的，但仅限于在一些特殊的场合使用，例如，新年、婚礼 |  |  |  |  |  |
| 是的，但仅限于在一些紧急情况下使用，例如身体不好，被当做最后的手段 |  |  |  |  |  |
| 是的，但是只有当野生动物数量足够多的时候 |  |  |  |  |  |
| 不会使用，如果野生动物受到灭绝的威胁或是保护物种 |  |  |  |  |  |
| 不会使用，野生动物有与人类共存的权利，它们从不应该因为人类的利益而被使用 |  |  |  |  |  |
| 我对这个问题的了解程度不够，不能很好的回答这个问题 |  |  |  |  |  |
| 无论哪种方式我都无动于衷 |  |  |  |  |  |

您有何其他宝贵意见，请详细写出

1. **下表列出了5种减少野生动物不可持续采伐的策略，请您对每种策略发挥的作用程度进行判断**

| **策略** | **有用** | **有一些影响** | **没用** |
| --- | --- | --- | --- |
| 加强立法监管和执法 |  |  |  |
| 利用社会压力转变人们使用野生动物产品的愿望 |  |  |  |
| 提供相当便宜的野生动物替代品 |  |  |  |
| 保护更多更多的物种，尽管这意味着限制使用这些物种 |  |  |  |
| 教育人们关于人畜共患病和与野生动物消费有关的其他健康问题的风险 |  |  |  |

若您有其他策略，请详细写出

**Part C：野生动物产品的替代品**

1. **请在下列关于野生动物替代品的陈述中，选出一条最符合您的观点的陈述：**

🞎 我在之前或是上个月曾经使用过几次野生动物产品的替代品

🞎 我对使用野生动物替代品具有丰富的经验，并且也将继续使用它们

🞎 我过去使用野生动物替代品的经历并没有让我很满意，因此未来我不会在使用它们。

相反，我将继续使用野外来源的野生动物产品

🞎 我至今没有用过任何野生动物替代品，但我很愿意尝试

🞎 我至今没有用过野生动物替代品，将来也不打算使用。我相信野生动物产品是不可替

代的

1. **如果您曾经使用过野生动物替代品，请具体写出：**
2. 您最近使用最多的野生动物替代品的名称：

1. 您使用的替代品的类型

🞎 养殖/抓获

🞎 与我平常使用不同的另一种野生动物

🞎 人工合成

🞎 家养的野生动物

1. 请写出您使用野生动物替代品代替而不使用野外来源的野生动物产品的原因：

1. **您是因为什么原因听说野生动物替代品的？请在下表中选出影响您对野生动物替代品态度的选项，并根据它对您的影响程度对您选中的选项进行排序（1代表影响程度最大，8代表影响程度最小）。未选中的选项不用进行排序。**

| **您熟悉野生动物替代品的原因** | **对您的影响程度（1-8）** |
| --- | --- |
| 🞎 教育 |  |
| 🞎 家人推荐 |  |
| 🞎 通过网络社交媒体 |  |
| 🞎 由中医从业者或药剂师推荐 |  |
| 🞎 由朋友或同事推荐 |  |
| 🞎 通过电视或广播 |  |
| 🞎 没有什么影响我，我自己找到的 |  |
| 🞎 对于野生动物替代品，我对他人的影响要大于别人对我的影响 |  |

若您有其他原因，请详细写出：

1. **请在下表列出的所有野生动物产品中，选出您愿意尝试其可用替代品的产品。并根据您尝试的可能性对选中的产品进行排序（1代表可能性最高，4代表可能性最低），未选中的产品不用进行排序。然后从下列的野生动物产品中选择一种，用X表示，并请您在附录中找到您标记为X的野生动物产品名称并进行相应问题的填写。**

| **野生动物产品（如果您愿意尝试其替代品，请打√）** | **可得到的替代品** | **您选择这种替代品的可能性** | **选择一种野生动物替代品，用X表示** |
| --- | --- | --- | --- |
| 🞎 自然毛皮 | 人造毛皮；仿造皮 |  |  |
| 🞎 鹿麝香 | 合成麝香；养殖麝香；植物替代品（石菖蒲）；动物替代品（麝鼠、麝牛） |  |  |
| 🞎 海产品 | 鲍鱼-植物替代品（蒟粉、魔芋） |  |  |
| 🞎 鹿茸 | 养殖；人参替代品；合成 |  |  |

若您有其他愿意尝试的野生动物替代品，请写出：

**Part E：受访者基本信息**

1. **性别：🞎**男 **🞎**女 **🞎**不想说
2. **年龄：**
3. **受教育程度：🞎**初中 **🞎**高中 **🞎**专科 **🞎**大学（请标出您的所在年级）：大一、大二、大三、大四 **🞎**研究生（请具体标出）：硕士研究生、博士研究生 **🞎**其他：
4. **专业（如果现在是个学生）：**
5. **家庭居住所在地（身份/直辖市）：**

**您的家乡属于：🞎**城市 **🞎**城镇或郊区  **🞎**农村

1. **父母职业**
2. **民族**
3. **职业：🞎**全职工作 **🞎**兼职工作 **🞎**全日制教育 **🞎**非全日制教育 **🞎**失业，找工作中 **🞎**失业，也没有找工作 **🞎**其他
4. **月收入：🞎**小于2000元 **🞎**2000-4000元 **🞎**4000-6000元 **🞎**6000-8000元 **🞎**8000-10000元 **🞎**大于10000元
5. **你有没有去过中国大陆以外的地方？ 🞎**是 **🞎**否
6. **您对本次调查问卷，还有什么宝贵的建议吗？**

1. **非常感谢您参与本次研究。如果您希望了解本研究的结果，请在下面写下您的电子邮件，我们会与您联系：**

**附录： 毛皮**

1. **您穿过或拥有过任何的毛皮制品吗？** 🞎是 🞎否
2. **您认为毛皮制品是？（请选择1个）**

🞎奢侈品 🞎必不可少的东西 🞎不重要

1. **什么场合适合穿皮草？**

🞎仅一些特殊场合，例如婚礼

🞎商业、专业活动

🞎寒冷的冬天

🞎没什么特殊场合是需要的

1. **请标明您对下列陈述的同意程度**
2. **野生动物的皮张不应该被制成皮草大衣**

🞎非常同意 🞎同意 🞎无所谓 🞎不同意 🞎非常不同意

1. **为了取皮繁育动物是合法的利用动物**

🞎非常同意 🞎同意 🞎无所谓 🞎不同意 🞎非常不同意

1. **天然毛皮的哪些特点对您最有吸引力？**

| **特点** | **非常重要** | **重要** | **无看法** | **没那么重要** | **一点都不重要** |
| --- | --- | --- | --- | --- | --- |
| 独特、稀有 |  |  |  |  |  |
| 野生捕获来源的毛皮更加真实 |  |  |  |  |  |
| 高价格体现了独特性和奢华品质 |  |  |  |  |  |
| 美学特征可以提高外在形象，时尚的象征 |  |  |  |  |  |
| 可收藏和可投资的物品 |  |  |  |  |  |
| 适合作为礼物 |  |  |  |  |  |
| 体现中国传统 |  |  |  |  |  |
| 耐用性 |  |  |  |  |  |

1. **您是否会考虑换用毛皮替代品？**
2. 养殖毛皮：🞎是 🞎否
3. 人工合成的毛皮：🞎是 🞎否
4. 不，野生毛皮是不可替代的，我只接受野生的真皮 🞎

**鹿麝香**

1. **您曾经使用或拥有过任何的鹿麝香产品吗？** 🞎是 🞎否
2. **您认为鹿麝香产品是？（请选择1个）**

🞎奢侈品 🞎必不可少的东西 🞎不重要

1. **您认为什么场合可以使用鹿麝香？**

🞎仅一些特殊场合，例如婚礼

🞎商业、专业活动

🞎没什么特殊场合是需要的

1. **请标明您对下列陈述的同意程度**
2. **麝香只有从野生动物品种中培育出来的才算正宗，但不一定非得是来源于麝鹿**

🞎非常同意 🞎同意 🞎无所谓 🞎不同意 🞎非常不同意

1. **麝的养殖应该是可持续的，即使这样做不能满足人们对麝产品的需求**

🞎非常同意 🞎同意 🞎无所谓 🞎不同意 🞎非常不同意

1. **龙涎香是一种来自抹香鲸的替代麝香。我会尝试使用这个，因为它的获得不会伤害野生动物物种**

🞎非常同意 🞎同意 🞎无所谓 🞎不同意 🞎非常不同意

1. **鹿麝香的哪些特性最吸引您？**

| **特点** | **非常重要** | **重要** | **无看法** | **没那么重要** | **一点也不重要** |
| --- | --- | --- | --- | --- | --- |
| 独特、稀有 |  |  |  |  |  |
| 野生捕获来源的鹿麝香更加真实 |  |  |  |  |  |
| 高价格体现了独特性和奢华品质 |  |  |  |  |  |
| 美学特征可以提高外在形象，时尚的象征 |  |  |  |  |  |
| 可收藏和可投资的物品 |  |  |  |  |  |
| 适合作为礼物 |  |  |  |  |  |
| 体现中国传统 |  |  |  |  |  |

1. **您是否会考虑换用麝香替代品？**
2. 养殖麝香：🞎是 🞎否
3. 人工合成麝香：🞎是 🞎否
4. 野生物种替代，例如麝鼠，果子狸，麝牛：🞎是 🞎否
5. 植物替代品（石菖蒲）：🞎是 🞎否
6. 来自抹香鲸的龙涎香：🞎是 🞎否

**鲍鱼**

1. **您以前是否消费过鲍鱼？**🞎是 🞎否
2. **您认为鲍鱼产品是？（请选择1个）**

🞎奢侈品 🞎必不可少的东西 🞎不重要

1. **您认为什么场合可以使用鲍鱼产品？**

🞎仅一些特殊场合，例如婚礼

🞎商业、专业活动

🞎没什么特殊场合是需要的

1. **鲍鱼产品的哪些特性最吸引您？**

| **特点** | **非常重要** | **重要** | **无看法** | **没那么重要** | **一点也不重要** |
| --- | --- | --- | --- | --- | --- |
| 独特、稀有 |  |  |  |  |  |
| 野生捕获来源的鲍鱼更加真实 |  |  |  |  |  |
| 高价格体现了独特性和奢华品质 |  |  |  |  |  |
| 美学特征可以提高外在形象，时尚的象征 |  |  |  |  |  |
| 适合作为礼物 |  |  |  |  |  |
| 体现中国传统 |  |  |  |  |  |
| 味道 |  |  |  |  |  |

1. **您是否会考虑换用鲍鱼替代品？**
2. 人工养殖：🞎是 🞎否
3. 替代海产品（没有那么稀有的）：🞎是 🞎否
4. 植物替代品（魔芋）：🞎是 🞎否

**鹿茸**

1. **您是否使用或拥有过任何鹿茸产品？**🞎是 🞎否
2. **您认为鹿茸产品是？（请选择1个）**

🞎奢侈品 🞎必不可少的东西 🞎不重要

1. **您认为什么场合可以使用鹿茸产品？**

🞎仅一些特殊场合，例如婚礼

🞎商业、专业活动

🞎没什么特殊场合是需要的

1. **鹿茸产品的哪些特点最吸引您？**

| **特点** | **非常重要** | **重要** | **无看法** | **没那么重要** | **一点都不重要** |
| --- | --- | --- | --- | --- | --- |
| 独特、稀有 |  |  |  |  |  |
| 野生捕获来源的鹿茸更加真实 |  |  |  |  |  |
| 高价格体现了独特性和奢华品质 |  |  |  |  |  |
| 美学特征可以提高外在形象，时尚的象征 |  |  |  |  |  |
| 可收藏和可投资的物品 |  |  |  |  |  |
| 适合作为礼物 |  |  |  |  |  |
| 体现中国传统 |  |  |  |  |  |
| 药用价值 |  |  |  |  |  |

1. **您是否会考虑使用鹿茸替代品？**
2. 人工养殖：🞎是 🞎否
3. 人参替代品：🞎是 🞎否
4. 合成替代品：🞎是 🞎否
